# Inorganic phosphate in growing calcium carbonate abalone shell suggests a shared mineral ancestral precursor

**DOI:** 10.1038/s41467-022-29169-9

**Published:** 2022-03-21

**Authors:** Widad Ajili, Camila B. Tovani, Justine Fouassier, Marta de Frutos, Guillaume Pierre Laurent, Philippe Bertani, Chakib Djediat, Frédéric Marin, Stéphanie Auzoux-Bordenave, Thierry Azaïs, Nadine Nassif

**Affiliations:** 1 Sorbonne Université, CNRS, Collège de France, Laboratoire de Chimie de la Matière Condensée de Paris (LCMCP), 4 place Jussieu, F-75005 Paris, France; 2 Laboratoire de Biologie des Organismes et Ecosystèmes Aquatiques (BOREA), Muséum National d’Histoire Naturelle/CNRS/IRD/Sorbonne Université/UCN/UA, Station marine de Concarneau, 29900 Concarneau, France; 3 Laboratoire de Physique des Solides (LPS), CNRS UMR 8502, Université Paris Saclay, F-91405 Orsay, France; 4 Laboratoire de RMN et Biophysique des Membranes, CNRS UMR7177, Université de Strasbourg, 4 rue Blaise Pascal, 67008 Strasbourg, France; 5 Muséum National d’Histoire Naturelle, UMR CNRS 7245, Bâtiment 39, CP 39, 57 rue Cuvier, 75231 Paris, France; 6 Laboratoire Biogéosciences, UMR CNRS 6282, Université de Bourgogne - Franche-Comté (UBFC) - 6, Boulevard Gabriel, 21000 Dijon, France

**Keywords:** Biomineralization, Solid-state NMR, Biomaterials

## Abstract

The presence of phosphate from different origins (inorganic, bioorganic) is found more and more in calcium carbonate-based biominerals. Phosphate is often described as being responsible for the stabilization of the transient amorphous calcium carbonate phase. In order to specify the composition of the mineral phase deposited at the onset of carbonated shell formation, the present study investigates, down to the nanoscale, the growing shell from the European abalone *Haliotis tuberculata*, using a combination of solid state nuclear magnetic resonance, scanning transmission electron microscope and spatially-resolved electron energy loss spectroscopy techniques. We show the co-occurrence of inorganic phosphate with calcium and carbonate throughout the early stages of abalone shell formation. One possible hypothesis is that this first-formed mixed mineral phase represents the vestige of a shared ancestral mineral precursor that appeared early during Evolution. In addition, our findings strengthen the idea that the final crystalline phase (calcium carbonate or phosphate) depends strongly on the nature of the mineral-associated proteins in vivo.

## Abstract (web summary)

Phosphate involvement in calcium carbonate biominerals raises questions on biomineralisation pathways. Here, the authors explore the presence of phosphate in the growing shell of the European abalone and suggest a shared mixed mineral ancestral precursor with final crystal phase being selected by mineral-associated proteins.

## Introduction

Biominerals are widespread in the tree of life and are found in many living organisms ranging from bacteria and archaea to eukaryotes^1,2^. In this latter domain, they are produced by protists^2^, fungi^3^, plants (e.g. cystoliths^4,5^, trichoms^6^), and numerous metazoans^7^ including sponges, cnidarians, brachiopods, bryozoans, molluscs, annelids, arthropods, echinoderms, and chordates. Biominerals are hybrid materials characterized by a subtle interplay between organic and inorganic phases. They exhibit emergent properties (mechanical resistance, optical properties) that are clearly distinct from that of their isolated constituents. Although the organic matrix displays numerous functions, most of which are poorly understood, it is usually described as a scaffold for the mineral deposition, by acting as a template for the nucleation^8–13^ and the subsequent crystal growth^14,15^. It is supposed to control biomineral size and morphology.

Chitin and collagen are, after cellulose, among the most abundant biopolymers on Earth^16^. Both are consistently associated to the biomineralization of calcium salts, namely calcium carbonate and calcium phosphates. Both are considered as bi- or tri-dimensional structuring organic substrates on which minerals deposit. Interestingly, among metazoans, one observes a general tendency to a partition between these two polymers: chitin is predominantly associated to calcium carbonate biominerals (calcite, aragonite, and vaterite) of protostomian exoskeletons (mollusc shell, arthropod cuticle) while collagen is mainly found in calcium phosphate (hydroxyapatite) endoskeletons of chordates^17^. Such specific organic-inorganic combination in living organisms seems to be a general principle. However, this principle suffers several exceptions.

It is assumed that collagen appeared after chitin in the Evolution, being present only in the metazoan kingdom, while chitin is also abundant in fungi phyla^18^. Nevertheless, the presence of collagen in some fungi suggests that this protein may have evolved from a common ancestor that existed before the divergence of fungi and animals^18^. Noticeably, biomineralized tissues composed of chitin and calcium phosphate are found in some species. In linguliform brachiopods—usually considered as living fossils— chitin is associated to hydroxyapatite although collagen is also present^19–21^. Analysis of the *Lingula* brachiopod shell reveals that the crystallographic match between chitin and apatite could be induced by an epitaxial growth of the mineral at the chitin surface^20^. This observation was further supported by in vitro experiments showing the oriented precipitation of apatite crystals in *β*-chitin without any additive proteins^22^. Other examples taken among the crustacean world illustrate the presence of inorganic phosphate (crystallized or amorphous) in chitin-/calcium carbonate-based biominerals: a biphasic mineral system consisting of hydroxyapatite and a mixture of calcium phosphate and carbonate was described in the cuticle of the marine peacock mantis shrimp *Odontodactyllus scyllarus*^23^; amorphous calcium phosphate (ACP) was identified in the lobster *Homarus americanus* cuticle, together with an amorphous calcium carbonate (ACC)^24^ but also in the edible crab *Cancer pagurus* calcitic exoskeleton^25^; finally, inorganic and bioorganic (*i.e*. metabolite) phosphates were both detected in the gastroliths of the crayfish *Cherax quadricarinatus*, enabling the stabilization of these calcium storage biominerals in the more soluble and non-crystalline ACC form^26^.

Phosphorus is also found in the mineralizing protist world, first as inorganic phosphate anions incorporated into the calcite without disrupting the structure^27^, similarly to Mg^2+^ in some calcified-biomineralized systems^28^ and secondly as element of the organic mineral-associated molecules like phosphorylated proteins, metabolites (phosphoenolpyruvate) and polyphosphates, used as ACC stabilizing agent. In the ectoplasmic plates of the alveolate protist *Coleps hirtus*, phosphorus and calcium colocalize but are correlated with the organic part of the alveolar plates^29^. Phosphorus is also found within a pool of intracellular calcium in the coccolith-forming *Emiliania huxleyi* haptophyte algae^30^. Conversely, among vertebrates, the most common substitution in bone apatite is carbonate, with type I collagen being the main component of the organic matrix. From the observation of calcareous sponges, a non-conventional mineralization process for vertebrate was proposed based on transient amorphous Ca‐carbonate bio‐seeds^31^. Regardless of whether it occurs, it is worth mentioning that otoliths consisting in calcium carbonate are found in the vestibular labyrinth of the vertebrate ear,^10^ *i.e*., in a collagenous environment. At last, the avian eggshell combines also calcite crystals deposited on a collagen-based membrane^32^.

Such exceptions in the dichotomy between calcium carbonate/chitin and calcium phosphate/collagen systems show that a clear-cut is not so obvious and that Evolution has forged unsuspected underlying crossed links between calcium phosphate, calcium carbonate, chitin and collagen worlds.

Little is known about the involvement of phosphate in controlling the biomineralization pathway of calcium carbonate-based systems but recent in vitro studies have contributed to shed light on its possible pivotal role. Strikingly, Ca/P/CO_3_ ions can coexist in a well-defined range of concentration as shown by a ternary diagram published few years ago^33^, where the carbonate/phosphate ratio is crucial to precipitate a given phase. In the same way, it was shown that ACC crystallizes into vaterite, aragonite or calcite depending on the phosphate concentration^34^, In fact, the phosphate-water interplay appears to tune ACC metastability, which either spontaneously transforms or is stabilized at ambient conditions^35^. Finally, phosphate ion was reported to reduce significantly the particle size of ACC^36^.

In the light of such steadily growing list of examples reporting on the co-localization of calcium carbonate and phosphate (including the carbonated-hydroxyapatite in bone and teeth), the coexistence of the three constituents, *i.e*., Ca/P/CO_3_, points toward the possible existence of a shared biomineral ancestral precursor that appeared early in Evolution.

In this work, we investigate the mineral phase of the larval shell from the European abalone *Haliotis tuberculata* (Mollusca, Vetigastropoda) to test further this hypothesis. Scanning transmission electron microscope (STEM) imaging combined with spatially-resolved electron energy-loss spectroscopy (EELS) down to the nanoscale and solid state nuclear magnetic resonance (ssNMR) were used to specify the mineral phase deposited in the early stages and study how the mineral components (Ca, P, and CO_3_) interact along the process of shell formation. Additional in vitro assays were conducted using a bioinspired pathway with different ratios of Ca/P/CO_3_ ions to determine their respective role in CaCO_3_ precipitation. Our results converge to the existence of a first-formed mineral phase, which combines calcium, phosphate, and carbonate, and possesses structural similarities with ACP. This suggests that specific P/CO_3_ ratio are selected through complexation with specific proteins of biological tissues determining the nature of the resulting mineral phase as previously proposed in the literature.

## Results and discussion

### Solid state NMR analysis of phosphate species in abalone larvae

**Fig. 1:**
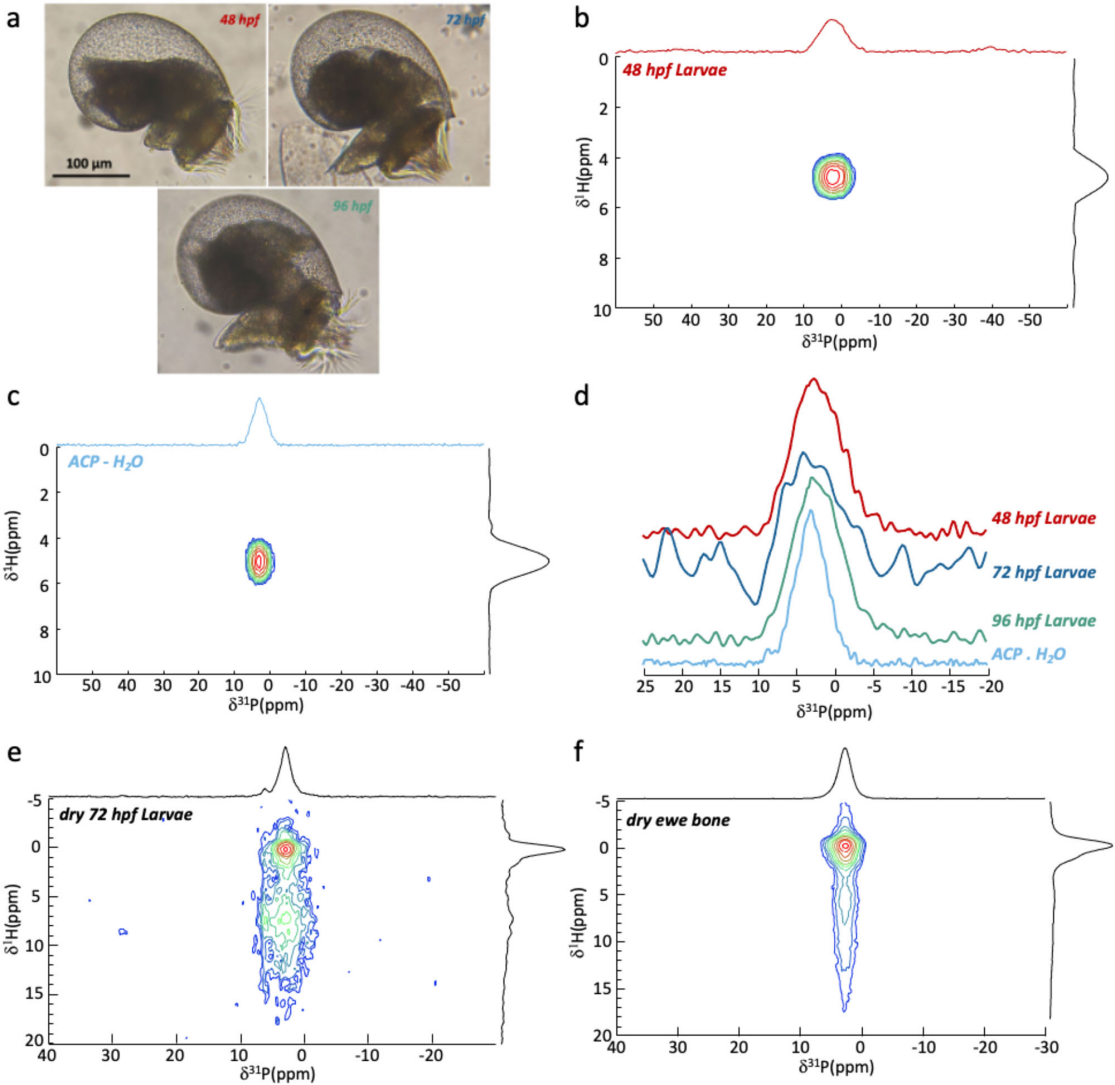
Identification of and ACP-like phase in fresh hydrated *H. tuberculata* larvae. **a** Optical microscopy images of *H. tuberculata* at different stages of development: 48, 72, and 96 hpf. ^1^H-^31^P HetCor spectra of **b** fresh *H. tuberculata* larvae at 48 hpf and **c** hydrated synthetic ACP (ACP.H_2_O). **d** Comparison of the ^1^H-^31^P HetCor extracted ^31^P rows of *H. tuberculata* and ACP.H_2_O. ^1^H-^31^P HetCor spectra recorded for **e** dry 72 hpf *H. tuberculata* larvae and **f** dry ewe bone.

*H tuberculata* is a marine gastropod of economic interest and a model for studying basic mechanisms of mollusc shell formation which is composed of aragonite and a variable amount of ACC at the early stages of development^37^ similarly to other molluscs^38,39^. Three freshly collected larval stages (48, 72, and 96 h post fertilization (hpf), Fig. 1a) were analyzed by solid state NMR without any chemical or thermic treatment to probe larvae in their native hydration state. Two-dimensional (2D) ^1^H-^31^P Cross Polarization MAS heteronuclear correlation (HetCor) experiments were used to edit rigid phosphorus-bearing species in close proximity with protonated species.

For all samples (Fig. 1b and Supplementary Fig. 1), the 2D ^1^H-^31^P HetCor spectra displays a strong and broad correlation resonance centred at δ(^31^P) ≈ 2.3 ppm (LW(^31^P) ≈ 7.8 ppm) and δ(^1^H) ≈ 4.8 ppm (although the resonance at δ^1^H ≈ 7.4 ppm in the 72 hpf sample is probably due to partial dehydration). According to both ^31^P and ^1^H chemical shifts, the phosphorous species involved correspond to phosphate species in close proximity with water molecules (free inorganic phosphate ions are not detected through CP due to their dynamic). According to the ^31^P chemical shift, the detected signal might be due to phosphate ions associated with calcium ions as the δ(^31^P) range from 5 to 0 ppm corresponds to mineral phases such as calcium orthophosphate (Supplementary Fig. 2). Moreover, the broad line width suggests highly disordered species. Taking also into account the strong correlation with water molecules, the correlation signal reminds similar experiments conducted on hydrated disordered calcium phosphate phase^15^.

2D ^1^H-^31^P HetCor spectra of a synthetic hydrated ACP presents a similar fingerprint (Fig. 1c). Comparison between the extracted ^31^P rows of biological and synthetic samples (Fig. 1d) shows that while the chemical shifts are similar, the linewidth is broader (LW ≈ 7.8 ppm) for the larvae samples compared to hydrated ACP (LW ≈ 5.8 ppm). ^31^P resonance of larvae should not only be of inorganic nature since the ^31^P signals below 0 ppm observed for biological samples might correspond to organic-P (*e.g*., phospholipids). We note that ^31^P NMR spectral signatures observed in aragonite-based biominerals such as the shell of the green-lipped mussel *Perna canaliculus*^40^ and the skeleton of four scleractinian corals^41^ were proposed to be assigned to ACP-like phases. However, the corresponding ^31^P resonances were observed at slightly higher chemical shift (4–6.5 ppm).

These observations evidence a large amount of phosphorus in *H. tuberculata* larvae under the form of “rigid” and hydrated phosphate ions. Surprisingly, comparison with the synthetic sample suggests the presence of an ACP-like phase.

To further investigate the presence of an ACP-like phase in *H. tuberculata* larvae, 72 hpf larvae were dried and analyzed by ssNMR. The resulting ^1^H-^31^P HetCor spectrum is displayed in Fig. 1e. We evidence a strong evolution of the NMR spectrum compared to fresh samples. Indeed, two distinct correlation signals of similar chemical shift (δ^31^P = 3.2 ppm) are observed and correlate with two distinct sources of protons. The first correlates with a sharp peak at δ(^1^H) = 0 ppm and the second with a weaker resonance spanning from 5 to 15 ppm. The first correlation peak evidences the presence of hydroxyapatite; the δ(^1^H) = 0 ppm corresponding to hydroxyl anions. The second resonance from δ(^1^H) = 5 to 15 ppm corresponds to residual water and HPO_4_^2−^ ions^15^. Comparison with similar experiments conducted on dry bone (Fig. 1f) confirms the previous assignment as we observe similar signals related to bone mineral: (i) the crystalline hydroxyapatite core (δ(^1^H) = 0 ppm) and (ii) the disordered mineral surface composed of HPO_4_^2−^ ions and residual water. The ^31^P chemical shift at 3.2 ppm is a strong indication of the formation of carbonated hydroxyapatite as found in bone mineral. Surprisingly, these results evidence the presence of carbonated hydroxyapatite in *H. tuberculata* larvae after drying. This indicates the association of phosphate and calcium ions in living larvae under a hydrated amorphous form that turns into crystalline phase through dehydration. The broad signal spreading from δ(^1^H) = 5–15 ppm for dry larvae may correspond to residual biological ACP-like phase and/or amorphous surface of the apatite crystals generated by dehydration. Additionally, a thin weak resonance of unknown origin at δ^31^P = 6.4 ppm is visible on the ^1^H-^31^P HetCor spectrum of dry larvae.

### Probing phosphate-carbonate distances in larval shell

To localize this possible ACP-like phase in the shell of *H. tuberculata* larvae, we performed {^1^H}-^13^C-{^31^P} CP REDOR experiments on a fresh 72 hpf larvae sample and probed the spatial proximities between phosphate and carbonate ions from the larval shell. To edit specifically ^13^C signals from the mineral, we developed a method to enrich the larval shell in ^13^C based on incubation in seawater enriched with ^13^C-labelled bicarbonate (see the “Methods” section and Supplementary Fig. 3). This labelling with NMR-active isotopes is not new for unicellular organisms^42^ but has been rarely employed for metazoans; one striking example is the “heavy mouse” from Duer and co-workers^43^.

**Fig. 2:**
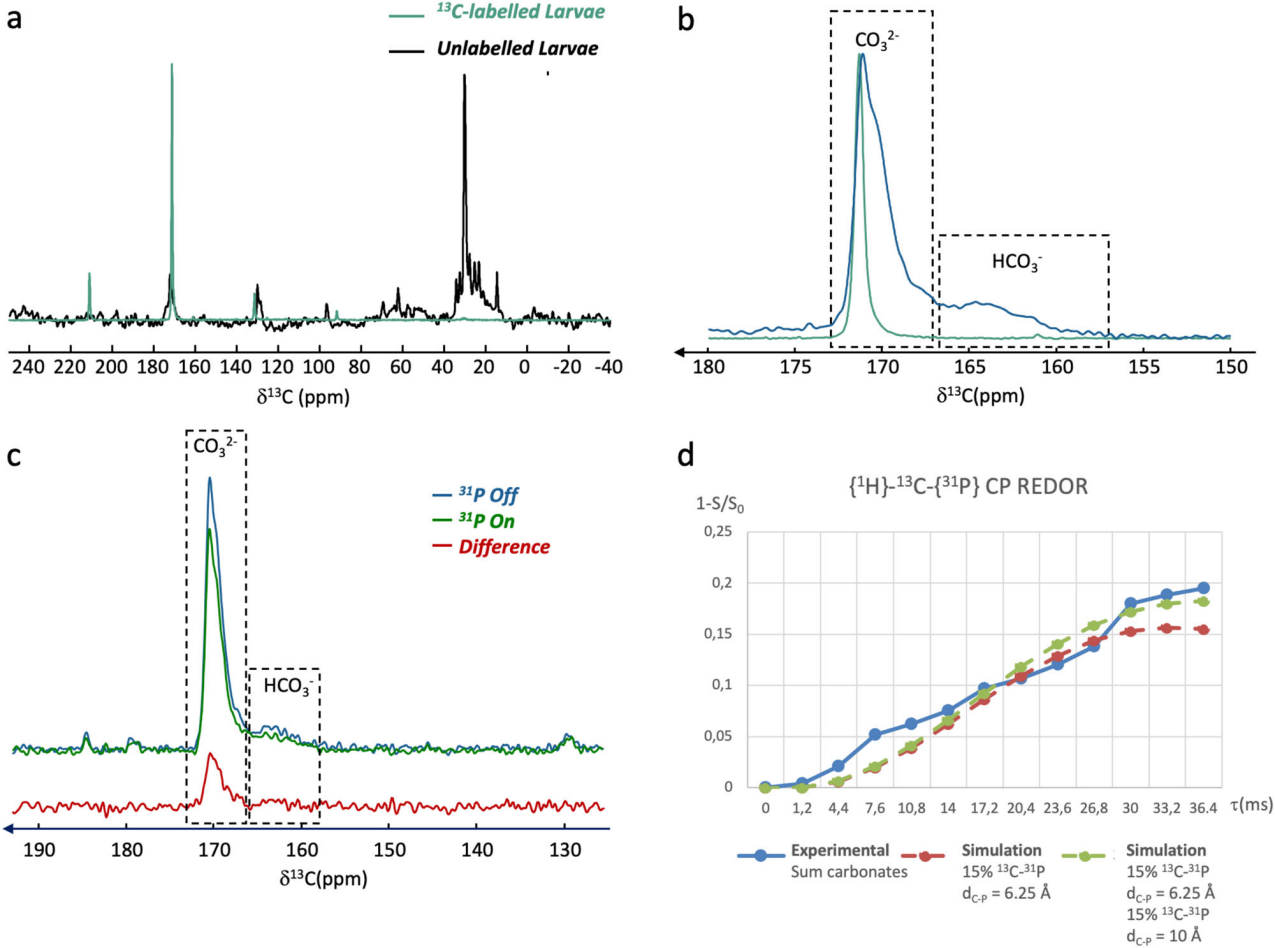
Localization of the ACP-like phase in abalone larval shell. **a** ^13^C DE MAS NMR spectra of unlabelled (black, NS = 620) and ^13^C-labelled (green, NS = 104) of fresh *H. tuberculata* larvae (72hpf). **b** Close-up on the carbonate region: ^13^C DE MAS NMR (green) and ^1^H-^13^C CP MAS NMR spectrum (blue, *t*_CP_ = 750 µs). **c** {^1^H}-^13^C-{^31^P} CP REDOR experiment of ^13^C-labelled fresh 72 hpf larvae. Recoupling time = 36 ms. **d** {^1^H}-^13^C-{^31^P} CP REDOR curve and numerical simulation (see text for details; error bars ±5%).

The efficiency of ^13^C enrichment method is highlighted in Fig. 2a which displays the ^13^C DE MAS NMR spectra (decoupled from protons) of unlabelled (black) and ^13^C-labelled (green) 72 hpf larvae. The ^13^C MAS NMR spectrum of unlabelled larvae displays resonances principally coming from lipids. Strikingly, the ^13^C MAS NMR spectrum of ^13^C-labeled larvae only displays a strong carbonate signal (spectra are normalized). We note that signals from the organic tissues are absent in these conditions. As the mineral fraction is quantitatively very low compared to the organic tissues, this experiment suggests that only the carbonates from the mineral fraction were enriched using this method. As such, we can conclude that a very large amount of carbonates involved in the shell formation comes from seawater. At these stages of development, it seems that larvae might not draw into their organic nutritional reserves to produce carbonates *via* enzymatic process but on the contrary, they may consume dissolved carbonates from seawater to build their shells.

Figure 2b displays a close-up on the carbonate region of the ^13^C DE MAS NMR spectrum of ^13^C-labelled 72 hpf larvae (green spectra). A unique resonance centred at 171 ppm is observed and corresponds to crystalline aragonite. Such observation is in agreement with previous FTIR analysis showing the presence of aragonite in *H. tuberculata*’s larval shell from 30 to 31 hpf^33,39^. Comparison with ^13^C MAS NMR spectrum of a synthetic sample highlights that biogenic aragonite from *H. tuberculata*’s larval shell at 72 hpf is similar in terms of linewidth to synthetic aragonite evidencing a high degree of crystallinity at such larval development (Supplementary Fig. 4). Figure 2b displays the ^1^H-^13^C CP MAS NMR spectrum of ^13^C-labelled 72 hpf larvae (blue spectra). Two types of signals are observed corresponding, according to their chemical shifts, to carbonates from 172 to 167 ppm and bicarbonates from 167 to 157 ppm. The presence of both carbonates and bicarbonates in the protonated environments of biogenic aragonite is also observed for mature nacre of *H. tuberculata*^44^. The full assignment was done on the basis of 2D ^1^H-^13^C HetCor experiment, variable contact time ^13^C CP MAS experiments and analysis of ^13^C chemical shift anisotropy (CSA) parameters obtained through slow spinning speed NMR experiments (Supplementary Fig. 5, Supplementary Fig. 6, Supplementary Table 3, and Supplementary Discussion 1). Thus, the larval shell is formed by crystalline aragonite together with a consequent amount of disordered carbonates and bicarbonates in protonated environment. According to previous studies on mature nacre, protonated carbonate species are proposed to be interfacial species in calcium carbonates shells^40,44^.

We performed {^1^H}-^13^C-{^31^P} CP REDOR experiments on a ^13^C-labelled 72 hpf larvae in order to probe the spatial proximities between phosphate and interfacial carbonates. The {^1^H}-^13^C-{^31^P} CP REDOR experiment is based on ^13^C-^31^P dipolar recoupling that enables here a selective identification of interfacial calcium carbonate close to phosphorus species. The REDOR experiment (Fig. 2c, recoupling time Rτ = 36.4 ms) is performed in two steps. First, a reference ^1^H-^13^C CP spectrum without dipolar recoupling is recorded (^31^P irradiation OFF; blue in Fig. 2c) then the reintroduction of the ^31^P-^13^C dipolar coupling is achieved (^31^P irradiation ON; green in Fig. 2c). The ^13^C resonances presenting an attenuation of intensity corresponds to carbon close to phosphorus species. The OFF-ON difference (red in Fig. 2c) shows a general attenuation for interfacial carbonates and to a lesser extent for bicarbonates species. The efficiency of the experiment is demonstrated by the absence of dephasing for aliphatic organic signals for the same recoupling time (Supplementary Fig. 7a).

Variation of the recoupling time allows the determination of C•••P distances through the numerical simulation of REDOR curves. The ^13^C resonances were decomposed and the fractions of signal amplitudes (1 − S/S_0_) were plotted against the recoupling time (Supplementary Fig. 7b–f). The signal intensities are disparate, but the global evolutions are similar and reach a maximum of dephasing of ∼0.2 (Rτ = 36.4 ms), suggesting that at least ∼20% of carbonates species (CO_3_^2−^ and HCO_3_^−^) are close to phosphate species. The full CO_3_^2−^ resonance evolution was investigated numerically. It turns out that simulation using 100% of single ^13^C-^31^P spin-pairs at a distance between 9 and 10 Å gives a correct final dephasing but fails to describe the rapid start of the curve (Supplementary Fig. 8). Numerical simulation gives a better result using 15% of ^13^C-^31^P spin-pairs at shorter distance of 6.25 Å (85% of carbonates without dephasing) but fails to describe the end of the curve. Finally, the best agreement is obtained using 30% ^13^C-^31^P spin-pairs at 6.25 Å (15%) and 10 Å (15%) (70% of carbonates without dephasing) (Fig. 2d). The REDOR analysis demonstrates that phosphates are located within larvae shell in the surrounding of the interfacial carbonates. Moreover, their repartition is heterogeneous as only ∼15–30% of these carbonates are close to phosphates and exhibit variable P•••C distances (from 6.25 to 10 Å). These distances are coherent with similar analysis conducted on synthetic^35^ and biologic samples^26^ where a homogeneous distribution of phosphates within the ACC matrix was evidenced but, conversely, our data suggest the presence of ACP-like domains that are adjacent to interfacial carbonates possibly in growing aragonite region.

### Ultrastructure and chemical composition of larval shells

**Fig. 3:**
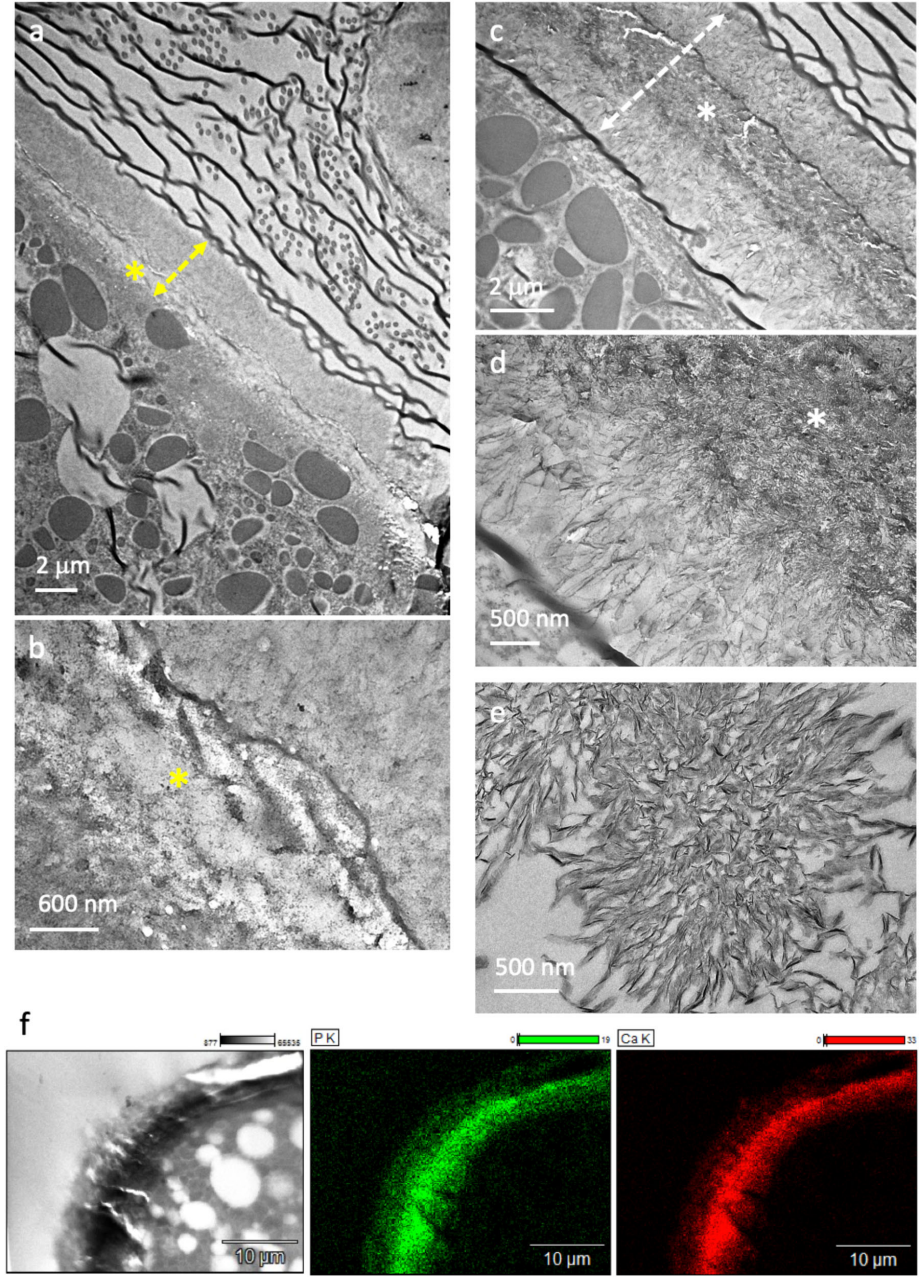
Ultrastructure and chemical composition of 48 hpf larval shell of *H. tuberculata*. TEM micrographs of the shell sections (double-headed dashed arrow) **a**–**d** at low magnification **a**, **c** showing two (**a**, **b**) or three (**c**, **d**) layers. Stars (*) show the less (yellow in a-b) and the more (white in **c**, **d**) contrasted layer. At higher magnification **b**, **d**, the presence of thin radial oriented particles reminding **e** in vitro spherulitic apatite observed in cross-section by TEM; **f** STEM-EDX analysis performed on the larval shell in cross-section showing the STEM micrograph of the analyzed region (left) and images from the related chemical analysis where the presence and the co-localization of phosphate (green) and calcium (red) is revealed.

TEM was performed on ultrathin sections of 48 hpf abalone larvae to observe the shell ultrastructure (Fig. 3). In Fig. 3a, the larval shell is observed in cross-section (doubled yellow dashed arrow) with the associated soft tissues that are mainly composed of lipidic vesicles (grey contrasted globules). The shell is about 5 µm in thickness but it may vary according to the shell orientation and between the shell areas chosen for TEM investigations. Two to three distinct structural layers are observed, and their presence is in agreement with previous scanning electron microscopy (SEM) investigations performed on fractured shells of mature veliger stages^45^. Two layers are most commonly observed (Fig. 3a, double-headed yellow dashed arrow) with one thick external layer (≃ 3 µm) and one lighter and thinner (<2 µm) inner layer (Fig. 3a, b, yellow star). However, we also observe shells composed of three layers (double-headed white dashed arrow, Fig. 3c), *i.e*. a more contrasted central layer (white star) surrounded by two lighter layers. They appear homogeneous in thickness (≃ 2 µm). For such shells, observations at higher magnification reveal the presence of thin radially oriented particles (Fig. 3d) in particular inside the layers that surround the central layer (white star). Surprisingly, they look like apatite spherulites seen in cross-section^46^ (Fig. 3e).

To go further, STEM-EDX analyses (Fig. 3f) were performed on these ultrathin sections. The results confirm the presence of phosphorus and its colocalization with calcium in the larval shell. This observation is in agreement with the NMR analyses showing that hydroxyapatite may precipitate inside the shell at an early stage of development upon drying indicating the presence of a Ca/P/CO_3_ ratio coherent with the precipitation of both calcium carbonate and phosphate. It remains unclear whether such artefactual apatitic spherulites result from either the TEM sample preparation or the vacuum in the TEM column since both may direct the crystallization of the amorphous mineral phase. Regardless, this observation evidences the strong influence of water content during the first steps of abalone shell formation. The fact that hydroxyapatite phase was revealed by NMR experiments performed on larvae without any treatment demonstrates that the Sorensen’s phosphate buffer is likely not the phosphorus source. It is also worth mentioning that the hypothesis of a calcium carbonate/phosphate amorphous phase stabilized by proteins was previously proposed for the cuticle of the lobster *Homarus americanus*, based on the precipitation of both hydroxyapatite and calcite^24^.

**Fig. 4:**
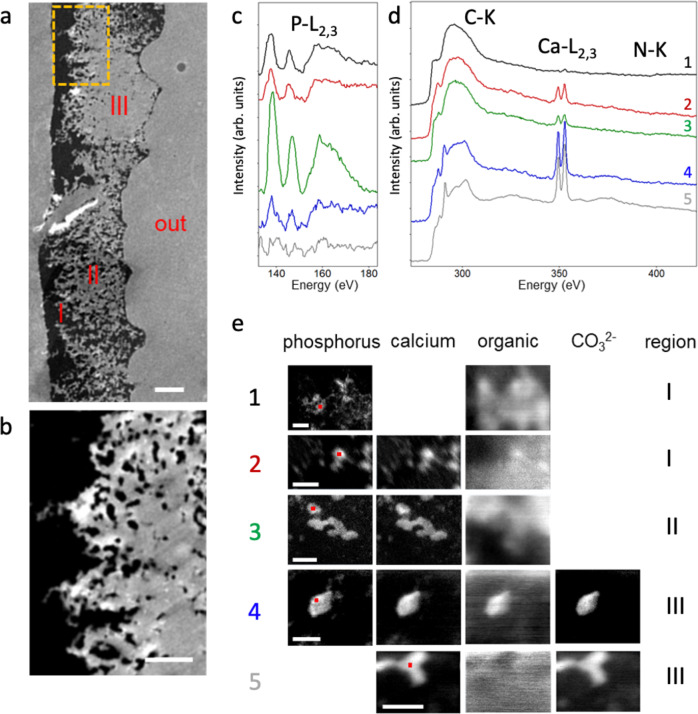
Chemical composition of 48 hpf larval by STEM-EELS. **a** HAADF image of an area across the 48 hpf larval shell of *H. tuberculata*. The outer part of the shell is indicated by “out”. **b** Magnified view of the part of the shell marked with a dashed orange rectangle on (**a**). **c** EEL spectra in the energy range corresponding to phosphorus (L_23_-edge), **d** carbon (K-edge), calcium (L_23_-edge) and nitrogen (K-edge) for the positions indicated by the red squares in the corresponding maps (1–5) in (**e**). The presented data sets correspond to the most common patterns observed in the sample. **e** Maps obtained from EELS data showing the distributions of phosphorus, calcium, organic species and carbonate in different areas across the larval shell. The region where each feature is commonly found is indicated by the labels I, II, and III in (**a**). Scale bar = 1 µm for (**a**), 0.5 µm for (**b**) and 50 nm for (**e**).

An in-depth study of the chemical composition of 48 hpf larval shell was performed by STEM-EELS for different areas across the protoconch (Fig. 4). The inner part of the shell was identified on STEM-HAADF images through the localization of the soft tissues (Supplementary Fig. 9 and Fig. 4a). The larval shell is not uniform over the transversal section and it appears to be brighter at the outside part. Note that the darkest contrast indicates that a part of the mineral was removed during the ultrathin-section preparation. Interestingly, a crenelated pattern located on the inner side (Fig. 4b) is observed, reminding the columnar arrangement in nacre^47^.

About 100 areas (dimensions ∼ 100 nm × 100 nm) were selected at different positions across the shell. The STEM electron probe was scanned over each area with 1 nm steps and a complete EEL spectrum was recorded at each position. The spectra corresponding to phosphorus, carbon, calcium, and nitrogen edges are presented in Fig. 4c, d for five different positions on the sample. Phosphorus and calcium were identified in EEL spectra by the double peak associated to their L_23_-edges, located at 138/147 and 349/352 eV respectively.

In bone apatite, the phosphorus L_3_-edge peak (~138 eV) presents a shoulder at ~141 eV that is also visible, to a lesser extent, for carbonated ACP (indicated by * in Supplementary Fig. 10c). This feature was attributed to the transition of a 2p electron from phosphorus to a 3d orbital in calcium. It has been observed in less soluble calcium phosphates (hydroxyapatite and α and β-Ca_3_(PO_4_)_2_, for instance) and tends to disappear when the phosphate ion is more loosely bound to calcium in the mineral lattice^48^. In *H. tuberculata* larval shell, the absence of the shoulder for the calcium phosphate deposits suggests that calcium is not strongly bound to phosphorus. This is compatible with the presence of high amounts of organic compounds associated with the calcium phosphate deposits.

The carbon K-edge is characterized by a broad peak around 301 eV and one or several narrow peaks at lower energy corresponding to C 1s → π* transitions. The position of the C 1s → π* peak varies with the C bonding environment. The maximum of the sharp peak was found at ∼285 eV for the epoxy resin, at ∼287–288 eV (with a smaller contribution at ∼285 eV) for organic species (proteins and chitin), and at ∼290 eV for the carbonate (see details in Supplementary Fig. 10b). EELS data do not allow the distinction between CO_3_^2−^ and HCO_3_^−^.

The elemental maps corresponding to the spatial distributions of phosphorus and calcium were obtained on each area from the EELS data set by integrating the intensity of their respective edges after background subtraction (Supplementary Discussion 2). The spatial distributions of the organic species and the carbonate were obtained by fitting the peaks associated to the C 1s → π* transition (details in Supplementary Discussion 2).

Our analysis reveals an important variability of the chemical composition depending on the region analyzed within the shell section. The results reported in Fig. 4 summarize the most common patterns found in 48 hpf larval shell of *H. tuberculata*. Phosphorus associated with organic compounds and no calcium (or only traces) (Fig. 4e, maps 1) was found in the strongly ramified inner regions (denoted “I” in Fig. 4a). Calcium deposits associated to phosphate were also found in these regions (Fig. 4d) but also in ramified middle regions (denoted “II” in Fig. 4a). Calcium carbonate deposits (Fig. 4e, maps 4 and 5) were only detected in outer regions “III” and in some cases, they were associated with small amounts of phosphate (Fig. 4e, map 4). In most cases, organic species were distributed in diffuse areas around calcium phosphate and calcium carbonate deposits (Fig. 4e, maps 1, 2, 3, and 5) but an increased organic concentration was found within some calcium deposits (Fig. 4e, map 4).

Overall, these results are consistent with the NMR data and support the presence of an unexpected calcium carbonate phosphate phase in abalone larval shell that appears to be present within the most inner part of the shell.

### In vitro mineralization of calcium carbonate in the presence of inorganic phosphate

To better understand the role of phosphate during calcium carbonate mineralization, we investigated in vitro the compositional evolution of minerals precipitated from aqueous solutions at different Ca/P ratios (from 0.3 to 6; Supplementary Table 1). The in vitro experimental conditions were inspired by the EELS data (Fig. 4 and Supplementary Fig. 10) where Ca and P colocalize in the very early stage of mineralization (*i.e.* inner part of the larval shell) while Ca and C (from CO_3_) are detected later on (*i.e*. outer part of the larval shell). The vapour diffusion method was preferred since it was used in the two-pioneer works demonstrating the involvement of mollusc-shell proteins in the resulting crystalline phase^49,50^^.^and it is a versatile approach to form either calcium carbonate^51–53^ or calcium phosphate^54–56^. H_2_KPO_4_ was added directly to the CaCl_2_ solution as a source of inorganic phosphate and the solutions were exposed to trigger mineral precipitation to a CO_2_(g) and NH_3_(g) atmosphere formed by (NH_4_)_2_CO_3_ decomposition.

**Fig. 5:**
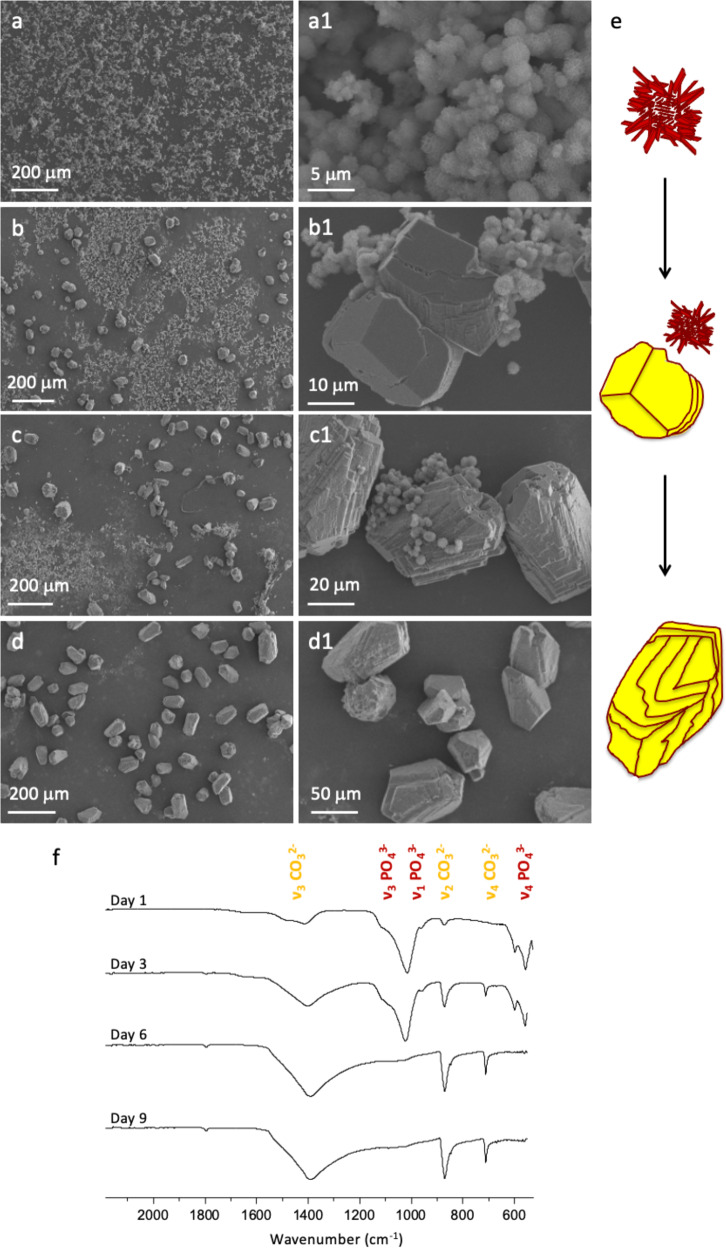
Temporal evolution of minerals formed in the presence of inorganic phosphate with Ca/P = 3. SEM images show the minerals formed after 1 day (**a**–**a1**), 3 days (**b**–**b1**), 6 days (**c**–**c1**), and 9 days (**d**–**d1**). The schematic representation of apatite evolution towards calcite with eroded surfaces is showed in (**e**). FTIR spectra of minerals formed after different reaction time (**f**).

SEM observations and FTIR analysis for the ratio Ca/P = 3 are presented in Fig. 5. Spherulitic hydroxyapatite particles are observed spreading on the glass surface together with few calcite crystals after 1 day (Fig. 5a, a1). The predominant formation of hydroxyapatite at the early stages of mineralization (days 1 and 3) is reflected by the higher intensity of the PO_4_^3−^ bands at ~1020 cm^−1^ (ν_3_, antisymmetric stretching), and 600–560 cm^−1^ (ν_4_), as compared to the CO_3_^2−^ bands (Fig. 5f). As the reaction evolves, the rhombohedral calcite becomes more evident, whereas the hydroxyapatite crystals tend to vanish (Fig. 5b–d1). Notably, calcite particles show eroded surfaces (Fig. 5c1) in agreement with previous observations showing that phosphate may interact with the faces of calcite crystals^57^. The gradual shift of hydroxyapatite to calcite is evidenced by FTIR. The intensity of the CO_3_^2−^ bands increases whereas the PO_4_^3−^ bands diminish as the reaction proceeds reflecting the conversion of calcium phosphate into carbonate. The switch between hydroxyapatite and calcite is clearly observed after 6 days of reaction by the absence of the PO_4_^3−^ bands. After 9 days, only big calcite crystals with an eroded surface precipitate (Fig. 5d, d1). It is likely that phosphate complexes with calcium ions, decreasing the supersaturation degree which in turn favours the formation of the thermodynamic product calcite. The composition of the biological tissue (including the HT’s physiological fluid) is much more complex than in our model, implying that specific organic molecules and other inorganic ions likely provide the polymorphism selection typical of each organism (here calcite *versus* aragonite). However, our model is of interest by its ability to illustrate the switch from calcium phosphate towards calcium carbonate.

A switch between hydroxyapatite and calcite is also observed for the other Ca/P ratios (*i.e*., 0.3, 1, and 6) (Supplementary Fig. 11) where the precipitation of calcite is delayed with an increase of the phosphate amount suggesting a competition between these two different phases. Here, the shift towards calcite occurs through a slow variation of pH and an increasing amount of CO_2_ that dissolves into the reaction medium (*versus* direct addition of carbonate), mimicking possibly the role of specialized organic components (*e.g*., proteins such as carbonic anhydrase)^58–60^. This is meaningful since the EELS data indicate that the appearance of CaCO_3_ takes place when it co-localizes with organic components (Fig. 4e). In vivo, this may occur through mixed precursor phases involving polyphosphates. Degradation of polyphosphates through enzymatic activity and pH change may lead to the presence of inorganic phosphate in the form of ACP or apatite in carbonate skeletons of crustaceans. The effect of pCO_2_ on the dissolution of HAP crystals might also interfere as previously shown^61^. Finally, phosphate ions may stabilize amorphous calcium carbonate phases through substitution of the carbonate and the formation of complexes with calcium^62–64^.

Overall, these results evidence that inorganic phosphate can be present at the early stages of calcium carbonate biomineralization in a Ca/P ratio that could lead to apatite precipitation. Although this result supports studies reporting on the deposition of mixed mineral phases during the calcification of organisms^30,65^, the present work does not stress on a crystalline apatite precursor phase. Strikingly, the Ca/P/CO_3_ ionic combination is reported in several organisms at different maturation stages, suggesting a common tract in biomineralization. Intracellular polyphosphate complexes were identified in calcium carbonate-based unicellular algae^66^. In coccolithophore algae, Ca^2+^ is translated from these phosphorus-rich pools to the mature mineral phase, strengthening the involvement of this mixed precursor phase in the early stages of calcification^67^. In such cases, specific enzymes are thought to be responsible for both directing the precipitation of calcite by introducing carbonate in these complexes and by hydrolyzing organic phosphates. Finally, mixed calcium phosphate and carbonate granules produced by osteoblasts were described as the mineral precursor of bone apatite through the hydrolysis of calcium-polyphosphate by alkaline phosphatase^65^.

Based on these results, we suggest that calcified organism controls biomineralization from a common precursor (with a specific Ca/P/CO_3_ ratio) that is driven towards calcium carbonate and/or phosphate structures owing to their own machinery comprising cells, physiological composition of precursor fluids, specialized polymers (proteins, polysaccharides, lipids) and metabolites. This may explain the presence of both phosphate and carbonate in mineralized organisms irrespective of the collagen/phosphate and chitin/carbonate specificity. From an evolutionary perspective, the widespread presence of intermixed mineral phases in living organisms that are taxonomically remote reinforces the idea that this mechanism may be general and shared by many organisms to form materials with superior mechanical properties and tailored features^68^.

The presence of a phosphate phase in carbonated species in a marine benthic organism is rather unexpected and intriguing, due in part to the fact that surface seawater is very depleted in phosphates^69^. Our finding goes along with observations made on other clades such as crustaceans where ACP was also described in their cuticle^25,70^ and diverse protists like coccolithophorids. It is also worth mentioning the hybrid hydroxyapatite/chitin shell of the lingulid brachiopods in which Watabe and Pan^71^ observed aggregates of apatite in the form of spherulites in the mineralized layer, presenting high similarity with our TEM observations.

The presence of this first-formed mineral phase, which combines calcium, phosphate, and carbonate (CaPCO_3_) in the larval shell of *H. tuberculata* may be related to the presence of alkaline phosphatase, detected in the mantle region of some bivalves and gastropods molluscs^72–76^ and also in the nacre of *Pinctada maxima*^77^. In *H. tuberculata* shell, an increase of the activity of this enzyme was reported and correlated with the strong shell growth phases^75^. The role of this ubiquitous hydrolase is to dephosphorylate macromolecules such as proteins and nucleic acids. In mammalians, this enzyme plays a role in the local concentration of inorganic phosphate species enhancing the precipitation of calcium phosphate^78^. In the same way, we can speculate that the CaPCO_3_ phase concentration in *H. tuberculata* shell may be regulated by the activity of an alkaline phosphatase.

In view of these different elements, the link between calcium carbonate and calcium phosphate species appears too entangled to be simply coincidental. The presence of ACP close to calcium carbonate in various shell species suggests the presence of a first-formed CaPCO_3_ phase at the early stages of the biomineralization shell process as speculated earlier for the lobster cuticle^24^. According to our in vitro study, different ratios between calcium, phosphate and carbonate cannot be excluded in the biogenic CaPCO_3_ phase.

The selection of the resulting aragonite phase might be governed by organic molecules (*e.g*. tissue-specific proteins selected during evolution) as described many years ago^49,50^. Nevertheless, the results from the in vitro analysis also suggest that the pH of the local environment might play a direct role in the selection of the resulting mineral phase.

This work demonstrates the presence of first-formed mineral phase which combines calcium, phosphate, and carbonate in the larval shell of the European abalone *Haliotis tuberculata*. This phase possesses some structural and chemical similarities with the hydrated amorphous surface layer of bone mineral. The presence of phosphate in a carbonated shell is intriguing and opens questions about its potential role in mollusc shell biomineralization.

The existence of an amorphous transient precursor of biomineralization applies to several metazoan lineages^4^, in particular, molluscs, arthropods, annelids, echinoderms, and vertebrates, suggesting that the cellular and molecular strategy of the 'amorphous precursor' pathway might have been already developed by an unmineralized Proterozoic ancestor at the base of the phylogenetic tree of metazoans^79^. Such a view would be in accordance with the old assumption of Lowenstam and Margulis that the regulation of intracellular calcium ion preceded (by far) the formation of calcified skeletons^80^. We suppose that the 'amorphous precursor' pathway was dictated by physiological constraints, *i.e.*, the need to detoxify the intracellular environment, *via*, for example, the formation of exocytosis vesicles containing amorphous inorganic granules^81^.

Our hypothesis points in the same direction by suggesting that an intermixed mineral precursor may be common to calcium-based biomineralized species, and might have formed and switched to either calcium carbonate or calcium phosphate final mineral phase under the control of organic molecules^49,50^. Our results underline once more the astonishing plasticity of calcium-based biominerals, a property that has contributed to their extraordinary evolutionary success across living systems.

## Methods

### Collection of abalone larvae

#### Fresh larvae sample

*Haliotis tuberculata* parental stock was collected on the northwest Brittany coast (Roscoff) and conditioned at the France-Haliotis abalone farm (48°36′50 N, 4°36′3 W; Plouguerneau, Brittany, France) in flowing seawater. Living larvae were obtained from controlled fertilizations and cultured in 350 L tanks at a seawater temperature of 17 ± 0.5 °C, leading to approximately 6 × 10^6^ living larvae. Larval growth and viability were evaluated under a binocular microscope (Leica, Germany) and the individuals were sampled at four key stages of the larval development (Fig. 1a): the trochophore stage (24 h post fertilization, hpf) characterized by the set-up of the larval shell (*i.e*. protoconch I), the post-torsional veliger (48 hpf) characterized by active calcification, the mature veliger stage (72 hpf) with the completed larval shell (protoconch II) and the pre-metamorphic veliger (96 hpf) which is the last stage of the pelagic life before larval settlement^45,82^. At each sampling time, about 5 L of seawater containing larvae were collected from the tank, filtered on a 40 µm-sieve and distributed into 15 ml tubes for analysis.

#### ^13^C-labelled larvae sample

24 to 72 hpf larvae are principally composed of organic tissues as the shell is very thin (about 3–5 µm in thickness) at this stage. In order to perform ^13^C NMR analysis of the calcium carbonate shell, we proceeded to a ^13^C enrichment of 72 hpf larvae shells as follows (Supplementary Fig. 3): first, around 100,000 24 hpf larvae were collected and incubated 48 h in seawater enriched with ^13^C-labelled NaHCO_3_ (NaHC*O_3_; 99% Aldrich) in 10 L fish tank. NaHC*O_3_ was added at the concentration of 2 mM (bicarbonate ions concentration in seawater) in order to double the natural concentration of bicarbonate in seawater. Then, larvae were collected and rinsed with filtered natural seawater and placed in 15 mL tubes filled with seawater. The seawater pH during the ^13^C-labelling experiment was monitored and was found to slightly decrease from 8.2 to 8.0. Larvae viability was regularly checked under a binocular microscope (Leica, Germany) and no particular increase in mortality was observed during the 48 h of incubation.

#### Dry larvae sample

72 hpf larvae shells were dried for 4 h under light vacuum (10 mbar) at room temperature.

### Bone samples preparation

#### Dry bone

Bone sample was harvested from two-year-old healthy French ewes. Bone was extracted from the proximal part of the diaphysis and distal epiphysis of humerus and femur. The work plan was reviewed and approved by the IMM Recherche’s Institutional Animal Care and Use Committee (IACUC) prior to the initiation of this study. The Animal Care and Use Committee of IMMR is registered at the CNREEA under the Ethics Committee n°37. The animal research centre (IMM-Recherche) received an agreement (n°75-14-01) by the “Direction Départementale de la Protection des Populations”. The studies were also performed in compliance with the Principles of Laboratory Animal Care, formulated by the National Society for Medical Research, and the Guide for the Care and Use of Laboratory Animals, by the Institute of Laboratory Animal Resources (published by the National Academy Press, Washington, D.C, 1996), as amended by the Animal Welfare Act of 1970 (P.L 91-579) and the 1976 amendments to the Animal Welfare were followed. The samples were dried for 24 h under sterile conditions, *i.e.* under laminar flow hood, at room temperature.

### Synthetic samples preparation

#### ACP synthesis

ACP was synthesized according to the Heughebaert method^51^. Briefly, Ca(NO_3_)_2_.4H_2_O (0.33 mol L^−1^) and (NH_4_)_2_HPO_4_ (0.33 mol L^−1^) were dissolved in aqueous solution of ammonia 1.10 and 0.50 mol L^−1^ respectively. Sequentially, 0.55 L of the calcium solution was then mixed with 1.30 L of the orthophosphate solution forming a precipitate which was immediately filtered through a Büchner funnel. The recovered precipitate was washed with ammonia aqueous solution (0.08 mol L^−1^), frozen in liquid N_2_, and lyophilized for a period of 72 h, and then stored at −20 °C.

#### Calcium carbonate and phosphate synthesis

The precipitations were performed using the vapour diffusion approach^83^ in the presence of different amounts of phosphate relying on a bioinspired pathway (aqueous solutions and room temperature)^54^. Before adding reactants, N_2_ was bubbled for 2 h in MQ water (1 L). Two flasks (30 mL) covered by punched Parafilm containing CaCl_2_ aqueous solution (20 mL; 0.01 mol L^−^^1^), and another flask (30 mL) containing fresh ammonium carbonate (2 g) were placed into a closed chamber (1000 cm^3^). To study the influence of phosphate on CaCO_3_ precipitation, 20 mL mixtures of CaCl_2_ (0.01 mol L^−1^) and H_2_KPO_4_ (0.01 mol L^−^^1^) were prepared, with Ca/P ratios ranging from 0.3 up to 6 (Supplementary Table 1). The solubilization of ammonia into the solution containing the precursor ions leads to the increase of pH thereby favouring the solubilization of CO_2_ and subsequently precipitation of minerals (calcium carbonate and/or carbonated hydroxyapatite). Glass slides were placed at the bottom of the flasks for further analysis. The reactions were conducted for 1, 3, 6, and 9 days. The reaction was stopped at different times and the pH measured using a pH metre. The glass slides were then removed from the flasks, carefully rinsed with ultrapure water (MilliQ) and absolute ethanol to remove the soluble salts.

### Solid-state nuclear magnetic resonance

The experimental conditions are described below and summarized in Supplementary Table 2. Different developmental stages (48, 72, and 96 hpf) of *H. tuberculata* larvae were investigated by ssNMR. Around 100,000 larvae at each stage were collected in 15 mL tubes filled with seawater. After 5 h (time needed to go from the hatchery to the NMR spectrometer), 4 or 7 mm diameter zirconia rotor were filled with fresh hydrated larvae and analyzed by magic angle spinning (MAS) ssNMR experiments without any sample preparation steps.

#### Two dimensional ^1^H-^31^P HetCor NMR experiments

Spectra of 48 and 96 hpf *H. tuberculata* larvae samples were recorded on a Bruker spectrometer operating at 11.74 Tesla using a 4 mm probe at a spinning frequency of *ν*_MAS_ = 8 kHz. Experimental parameters were the following: recycle delay RD = 2 s, contact time *t*_CP_ = 1 ms, number of scans NS = 400, number of *t*1 increments = 40. Spectra of 72 hpf *H. tuberculata* larvae and hydrated ACP (ACP.H_2_O) samples were recorded on a Bruker spectrometer operating at 7.05 Tesla using a 4 mm probe at *ν*_MAS_ = 8 kHz. Experimental parameters: RD = 2 s, *t*_CP_ = 1 ms, NS = 800, number of *t*1 increments = 40. Spectra of dry 72 hpf *H. tuberculata* larvae and dry ewe bone samples were recorded on a Bruker spectrometer operating at 7.05 Tesla using a 4 mm probe at *ν*_MAS_ = 14 kHz. Experimental parameters: RD = 3.5 s, *t*_CP_ = 10 ms, NS = 180, number of *t*1 increments = 100.

^*13*^*C direct excitation and* ^*13*^*C CP MAS NMR experiments*. ^13^C direct excitation (DE) NMR spectra of ^13^C-labelled and unlabelled 72 hpf *H. tuberculata* larvae samples were recorded on a Bruker spectrometer operating at 7.01 Tesla using a 7 mm probe at ν_MAS_ = 5 kHz. A radio-frequency pulse of 30° was used such that π/6(^13^C) = 1.1 µs. Experimental parameters: RD = 600 s, NS = 104-620 s. ^13^C CP MAS NMR spectrum of ^13^C-labelled 72 hpf *H. tuberculata* larvae sample was recorded on a Bruker spectrometer operating at 7.01 Tesla using a 7 mm probe at *ν*_MAS_ = 5 kHz. Experimental parameters: RD = 10 s, *t*_CP_ = 750 µs, NS = 10240. Variable contact time experiments were recorded using *t*_CP_ = 250, 500, and 750 µs, 1, 2, 5, 10 ms and NS = 3312 for each experiment. Slow MAS experiments were recorded at *ν*_MAS_ = 1.5 kHz and *t*_CP_ = 1 ms. Two dimensionnal ^1^H-^13^C HetCor NMR spectrum were recorded at *ν*_MAS_ = 5 kHz using the following parameters: RD = 7.5 s, *t*_CP_ = 750 µs, NS = 1440, number of *t*1 increments = 32.

{^1^H}-^13^C-{^31^P} CP REDOR NMR experiments on ^13^C-labelled 72 hpf *H. tuberculata* larvae sample were recorded on a Bruker spectrometer operating at 7.01 Tesla using a 4 mm probe at ν_MAS_ = 5 kHz. The temperature was controlled at 278 K in order to maximize the efficiency of the ^13^C-^31^P dipolar recoupling. The ^13^C magnetization is generated by a standard CP step, and then a rotor-synchronized refocusing pulse is applied on ^13^C channel. To allow the re-introduction of the ^31^P-^13^C dipolar coupling, dephasing ^31^P π pulses were applied at each rotor period during the ^13^C evolution. The radio-frequency fields were set such that π pulses π(^13^C) = π(^31^P) = 12 µs. Twelve experiments were recorded at various recoupling time: 1.2, 4.4, 7.6, 10.8, 14, 17.2, 20.4, 23.6, 26.8, 30, 33.2, and 36.4 ms. The following parameters were used: RD = 10 s, *t*_CP_ = 750 µs, NS = 2432. Data acquisition was done through an interleaved mode where a single S_0_ (without ^31^P irradiation) and a single S (with ^31^P irradiation) transients were collected alternatively in order to minimize the spectrometer instabilities. REDOR difference Δ*S* = *S*_0_ − *S* exhibits the ^13^C resonances dipolarily coupled to ^31^P, consequence of a spatial proximity between the two nuclei.

NMR spectra were acquired with Topspin 3.2 (Bruker) and were displayed with DMfit 2019. REDOR experiments were simulated using SIMPSON 4.2.1. and Excel 2019.

### Fourier transform infrared spectroscopy (FTIR)

FTIR spectra were recorded on a Brucker-Vector 22 FTIR spectrometer and displayed with OriginPro 9.1.

### Microscopies and related analysis

#### Light microscopy

Light microscopy was performed on the glass slides by using a transmission Zeiss AxioImager A2 POL, equipped with crossed polarizers and an AxioCam CCD camera.

#### Scanning electron microscopy (SEM)

Glass slides were covered by a 10 nm layer of gold and observed by SEM using a Hitachi S-3400N operating at 8 kV.

#### Ultrathin sections preparation for transmission electron microscopy (TEM) and scanning transmission electron microscopy (STEM)

Larval samples were chemically fixed with a solution composed (in v/v) of 2% glutaraldehyde, 2% paraformaldehyde, 0.1% picric acid in 0.09 mol L^−1^ Sorensen buffer (Na_2_HPO_4_ - pH 7.5) during 2 h at room temperature and placed one night at 4 °C. Then, the samples were rinsed twice with Sorensen buffer during 1 h for each time. The larvae were stored at 4 °C and then dehydrated through a graded series of ethanol and included in acrylic resin. Ultrathin sections were performed using an ultramicrotome (Reichert Ultracut) equipped with a diamond knife. The ultrathin sections (60 nm) were deposited on copper grids coated with a carbon film. Before analysis, the samples were contrasted by uranyl acetate. TEM observations were performed on a Hitachi H7700 operating at 80 kV.

#### Scanning transmission electron microscopy energy-dispersive X-ray spectroscopy (STEM-EDX) and scanning transmission electron microscopy electron energy loss spectroscopy (STEM-EELS)

The chemical composition of the larvae shell was analyzed by EDX and EELS. EDX data were recorded using STEM mode on Hitachi H7700 operating at 100 kV. HAADF (High-angle annular dark-field) images and EELS data were recorded using a STEM Vacuum Generators HB501 operated at 100 keV and equipped with a home-modified Gatan spectrometer. Samples were cooled down to approximately 170 K using a home-made cryo-stage to minimize beam radiation damage. Core-loss spectra were acquired with an energy dispersion of 0.35 eV/channel in the energy range corresponding to the characteristic edges for the elements of interest (P, C, Ca, and N). Typical energy resolution was about 1 eV and the beam diameter was estimated to 1 nm. Spectra were recorded in the so-called Spectrum Imaging mode^84^, *i.e*. the focused electron probe is scanned, step by step, over the region of interest, and a whole spectrum is acquired at each position. About 10000 spectra were recorded on each area. The energy calibration of the spectra and the data processing are detailed in the SI section (Supplementary Discussion 2).

### Statistics and reproducibility

Living larvae were collected from four different sampling campaigns (September 2015, October 2016, June 2016, and June 2017) at the France Haliotis abalone farm. Larvae were randomly chosen from the fixed developmental stages for light and electron-microscope observations. The micrographs displayed in the Figures are representative of the observations. Please note that micrographs shown in Fig. 1a were taken from similar oriented free-swimming larvae.

## Supplementary information

Supplementary Information

## Data Availability

All data generated or analyzed during this study are included in this published article (and its supplementary information files), and are also available from the corresponding authors on reasonable request.
