# Supplementary material for: Inorganic phosphate in growing calcium carbonate abalone shell suggests a shared mineral ancestral precursor
Source: Nat Commun. 2022 Mar 21;13:1496. doi: 10.1038/s41467-022-29169-9 (PMC8938516; doi:10.1038/s41467-022-29169-9)
Supplement: Supplementary file 1 — Supplementary Information [file 41467_2022_29169_MOESM1_ESM.docx]

**Supplementary Information**

**Inorganic phosphate in growing calcium carbonate abalone shell suggests a shared mineral ancestral precursor**

*Widad Ajili^1,2 †^, Camila B. Tovani^1†^, Justine Fouassier**^1^, Marta de Frutos^3^,* *Guillaume Pierre Laurent^1^,* *Philippe Bertani^4^, Chakib Djediat^5^, Frédéric Marin^6^, Stéphanie Auzoux-Bordenave^2^, Thierry Azaïs^1^, Nadine Nassif^1^**

^1^Sorbonne Université, CNRS, Collège de France, Laboratoire de Chimie de la Matière Condensée de Paris (LCMCP), 4 place Jussieu, F-75005, Paris, France.

^2^Laboratoire de Biologie des Organismes et Ecosystèmes Aquatiques (BOREA), Muséum National d'Histoire Naturelle/CNRS/IRD/Sorbonne Université/UCN/UA, Station marine de Concarneau, 29900 Concarneau, France.

^3^Laboratoire de Physique des Solides (LPS), CNRS UMR 8502, Université Paris Saclay, F-91405 Orsay, France.

^4^Laboratoire de RMN et Biophysique des Membranes, CNRS UMR7177, Université de Strasbourg, 4 rue Blaise Pascal, 67008 Strasbourg, France.

^5^Muséum National d'Histoire Naturelle, UMR CNRS 7245, Bâtiment 39, CP 39, 57 rue Cuvier, 75231 Paris, France.

^6^Laboratoire Biogéosciences, UMR CNRS 6282, Université de Bourgogne - Franche-Comté (UBFC) - 6, Boulevard Gabriel, 21000 Dijon, France.

† These authors contributed equally to this work

*Nadine Nassif.

**Email:** nadine.nassif@sorbonne-universite.fr

**Supplementary Table 1.** Summary of the experimental conditions used for the vapor diffusion calcium carbonate synthesis in presence or not of phosphate ions for different ration Ca/P and time of precipitation.

| **[Ca]**  **mmol/L** | **[PO_4_^3-^]**  **mmol/L** | **Ca/P** | **pH** | | | | | | |
| --- | --- | --- | --- | --- | --- | --- | --- | --- | --- |
|  |  |  | **Reaction Day** | | | | | | |
|  |  |  | **0** | **1** | **2** | **3** | **6** | **9** | **12** |
| 10 | - | - | 6.5 | 9.6 | 10.5 | 10.1 | 9.6 | 9.1 | 8.9 |
| 10 | 33 | 0.3 | 4.6 | 9.4 | 9.3 | 9.2 | 9.1 | 9.0 | 8.9 |
| 10 | 10 | 1.0 | 4.8 | 9.4 | 9.3 | 9.2 | 9.0 | 9.0 | 8.9 |
| 10 | 3.33 | 3.0 | 5.1 | 9.5 | 9.2 | 9.1 | 9.0 | 9.0 | 8.9 |
| 10 | 1.66 | 6.0 | 5.2 | 9.6 | 9.3 | 9.1 | 9.0 | 9.0 | 8.9 |

| Age (hpf) | State | NMR experimentS | NMR parameters |
| --- | --- | --- | --- |
| 48  72  96 | Fresh,  Hydrated | 2D ^1^H-^31^P HetCor | ν_MAS_ = 8 kHz  RD = 2 s  t_cp_ = 1 ms  NS = 400  Number t_1_ increments = 40 |
| 72 | Dry | 2D ^1^H-^31^P HetCor | ν_MAS_ = 14 kHz  RD = 3 s  t_cp_ = 10 ms  NS = 152  t_1_ increments = 160 |
| 72 | Fresh,  hydrated | ^13^C direct exitation MAS | ν_MAS_ = 5 kHz  π/6 pulse = 1.1 µs  RD = 600s  NS = 620 |
| 72 | ^13^C-labelled  Fresh hydrated | ^13^C direct exitation MAS | ν_MAS_ = 5 kHz  π/6 pulse = 1.1 µs  RD = 600s  NS = 104 |
|  |  | 1D ^13^C CP MAS | ν_MAS_ = 5 kHz  RD = 10 s  t_cp_ = 750 µs  NS = 10240 |
|  |  | 2D ^1^H-^13^C HetCor | ν_MAS_ = 5 kHz  RD = 7.5 s  t_cp_ = 750 µs  NS = 1440  t_1_ increments = 32 |
|  |  | {^1^H}-^13^C-{^31^P} CP Rotational-Echo Double-Resonance (CP-REDOR) | ν_MAS_ = 5 kHz  RD = 7.5 s  t_cp_ = 750 µs  Recoupling time = 1.2; 4.4; 7.6; 10.8; 14; 17.2; 20.4; 23.6; 26.8; 30; 33.2 and 36.4 ms  NS = 2432  T = 278 K |

**Supplementary Table 2.** *Haliotis tuberculata* larva samples and typical experimental NMR parameters.


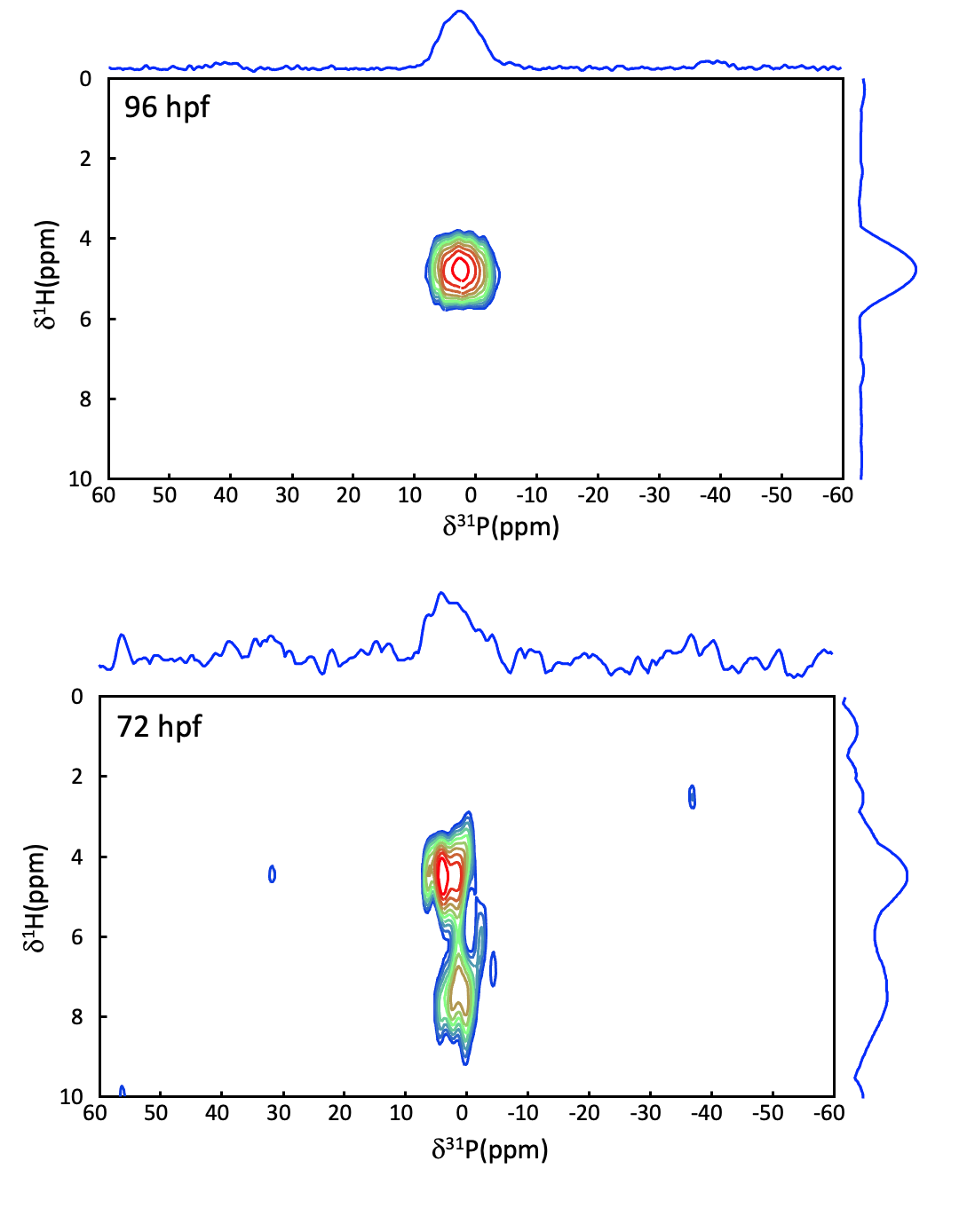


**Supplementary Figure 1.** Two dimensional ^1^H-^31^P HetCor spectra of fresh 72 and 96 hpf *H. tuberculata* larvae.

**
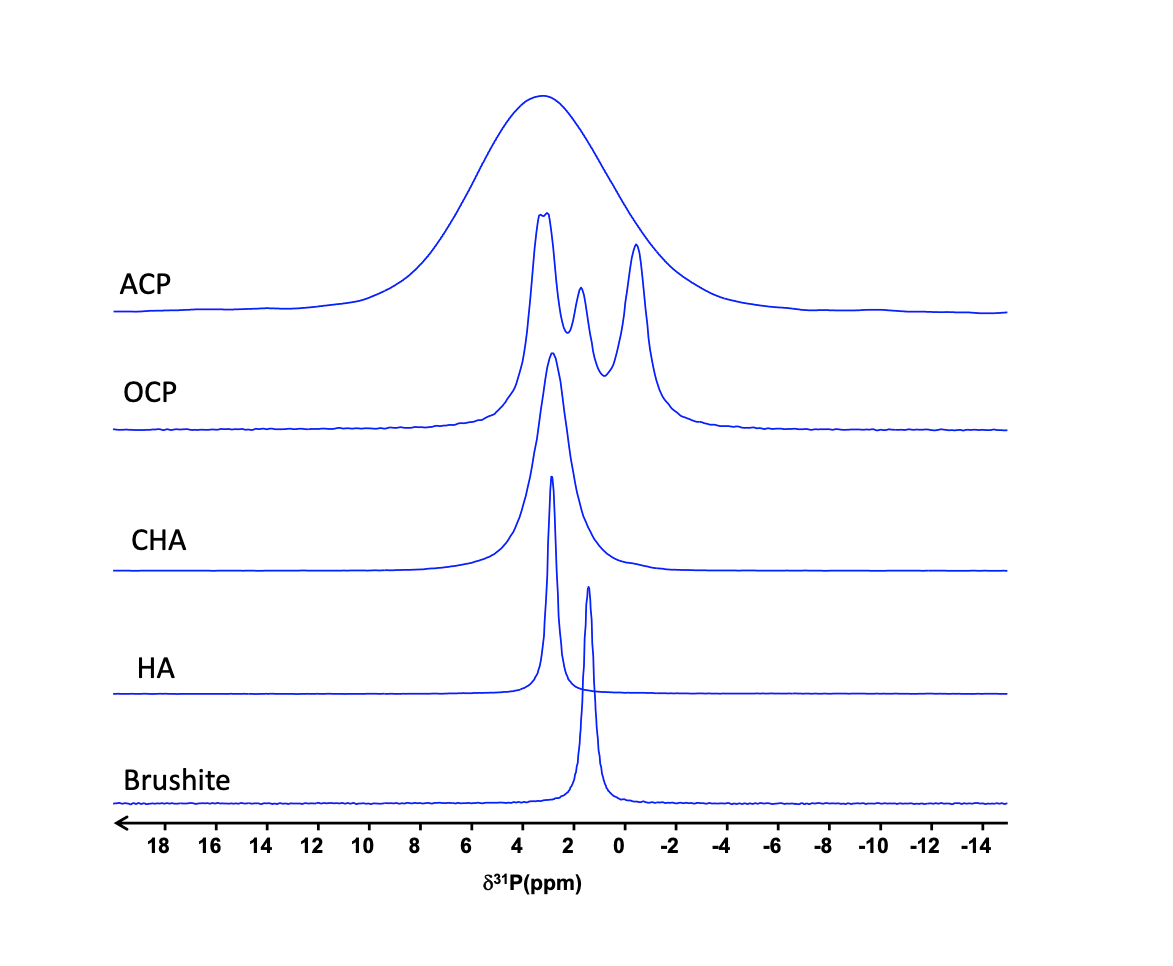
Supplementary Figure 2.** ^31^P MAS NMR spectra of various calcium phosphate phases (amorphous calcium phosphate (ACP), octacalcium phosphate (OCP), carbonated hydroxyapatite (CHA), stoichiometric hydroxyapatite (HA) and brushite) highlighting their ^31^P chemical shift range.

**
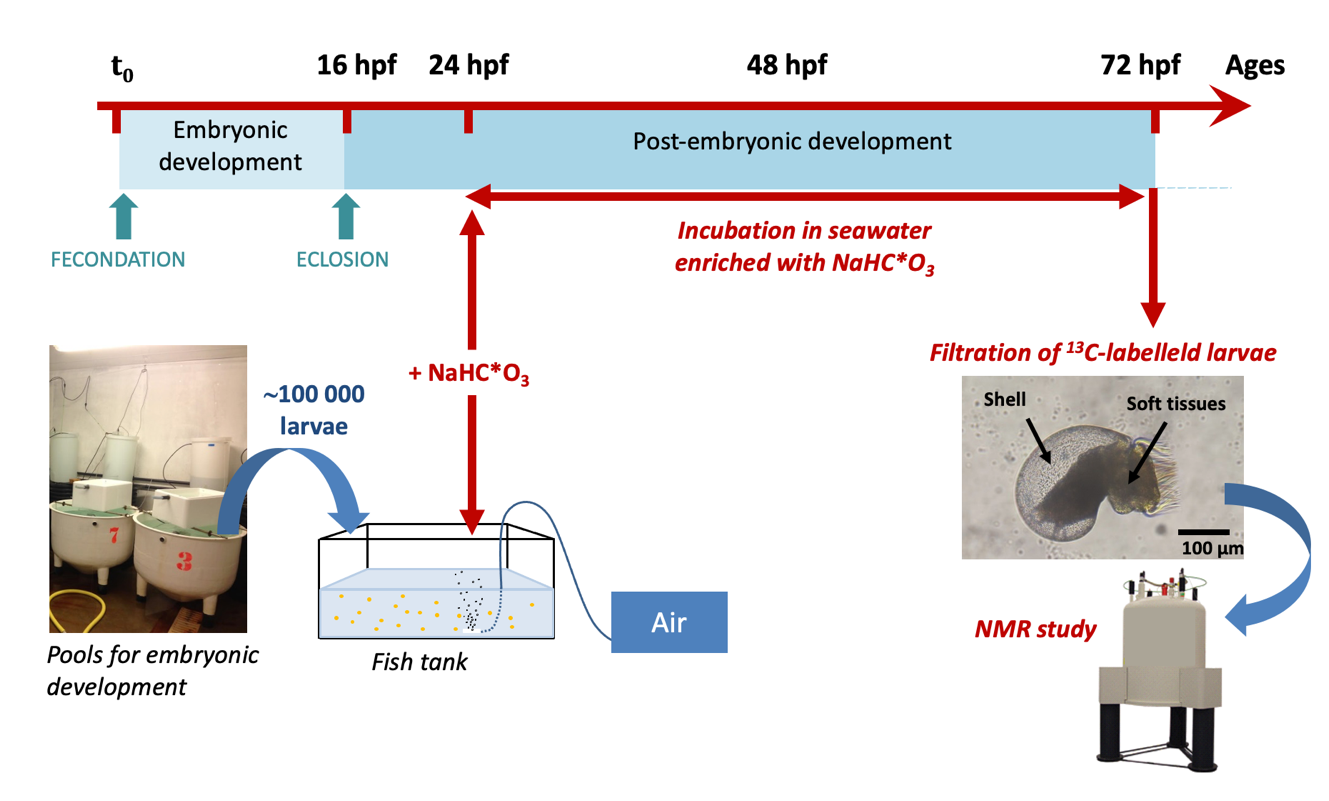
**

**Supplementary Figure 3**. Schematic representation of the set-up for ^13^C-labelling of *H. tuberculata* larval shells.


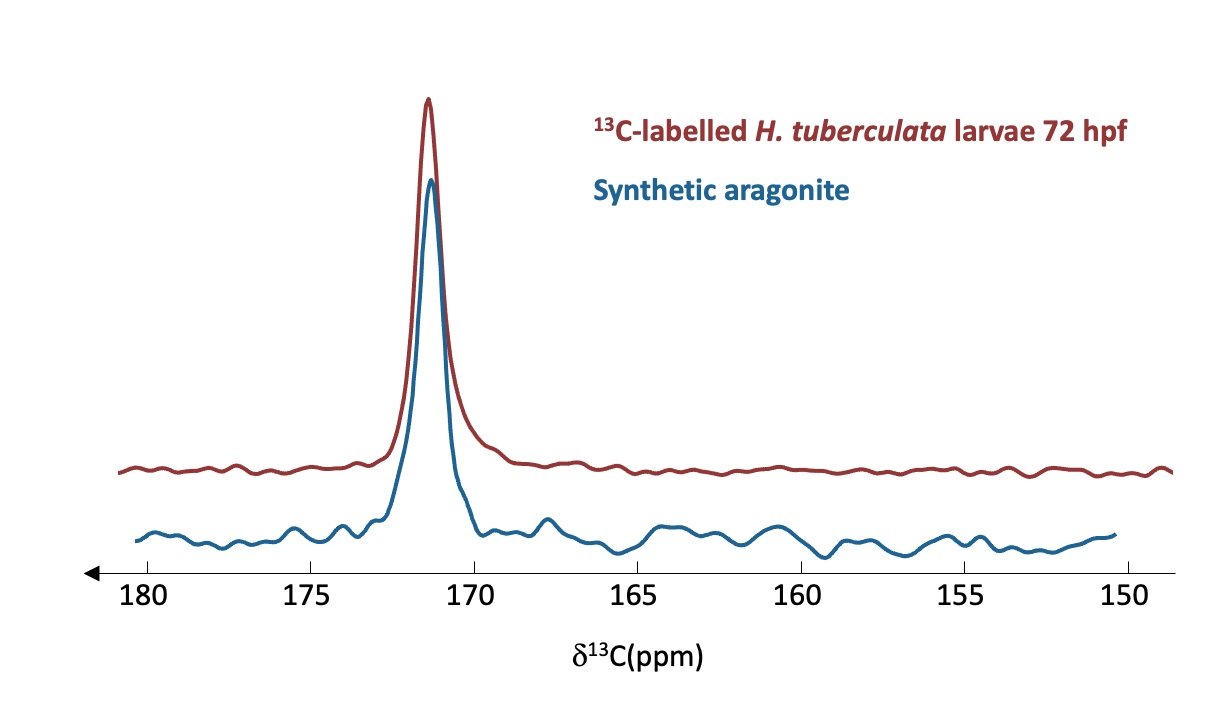


**Supplementary Figure 4.** Comparison of the ^13^C DE MAS NMR spectra of ^13^C-labelled *H. tuberculata* larvae (72 hpf) and synthetic aragonite.


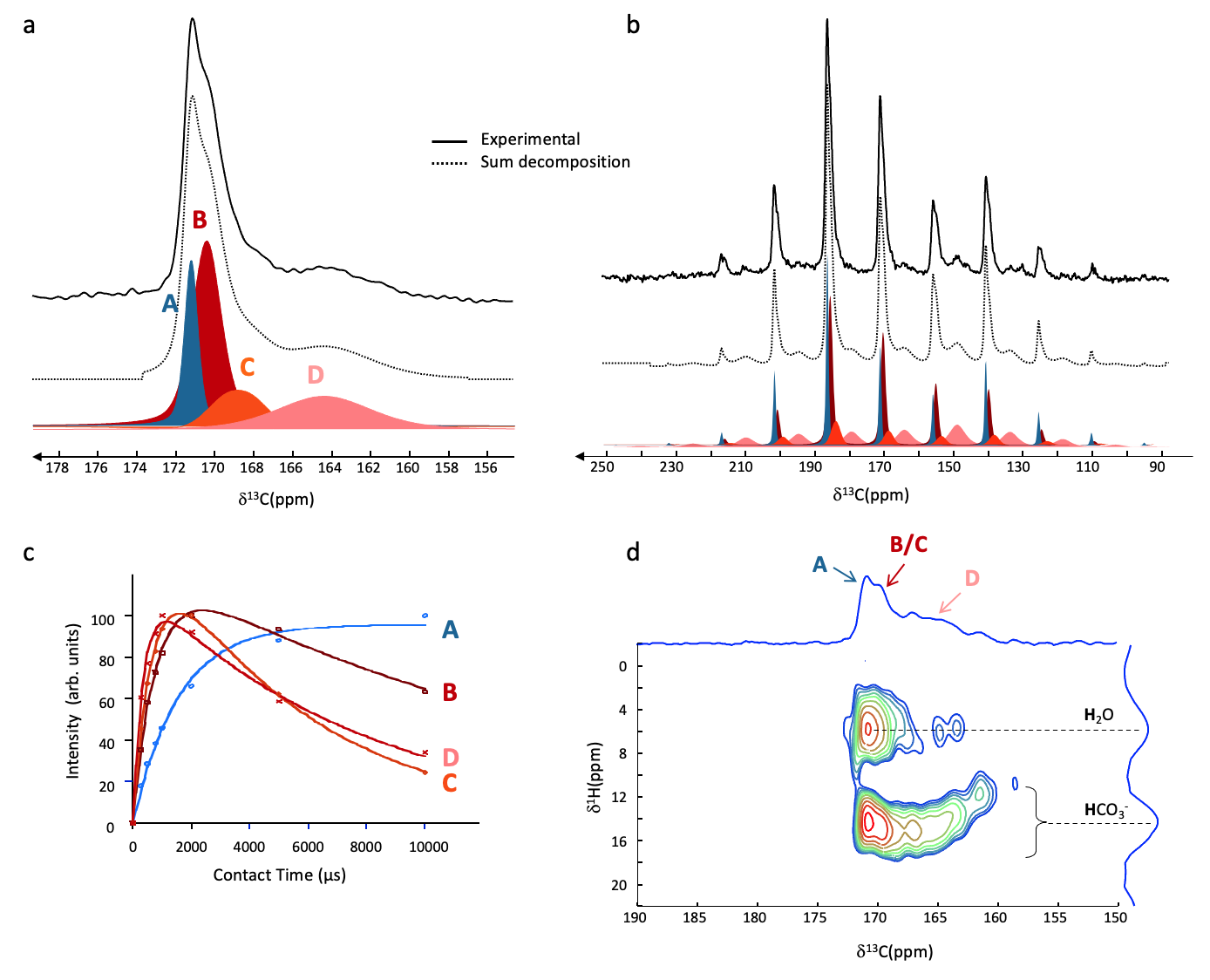


**Supplementary Figure 5.** (a) Decomposition of the ^13^C CP MAS NMR spectrum (t_CP_ = 750 µs) of ^13^C-labelled *H. tuberculata* 72 hpf larvae. (b) Slow spinning speed ^13^C CP MAS NMR experiment (ν_MAS_ = 1.5 kHz; t_CP_ = 1 ms) together with best fit of CSA parameters. (c) Variable contact time ^13^C CP MAS NMR experiments together with numerical simulation (normalized). (d) 2D ^1^H-^13^C HetCor NMR spectrum (t_CP_ = 1 ms). Chemical shifts assignment and NMR parameters extracted from these experiments are summarized in **Supplementary Table 3**.


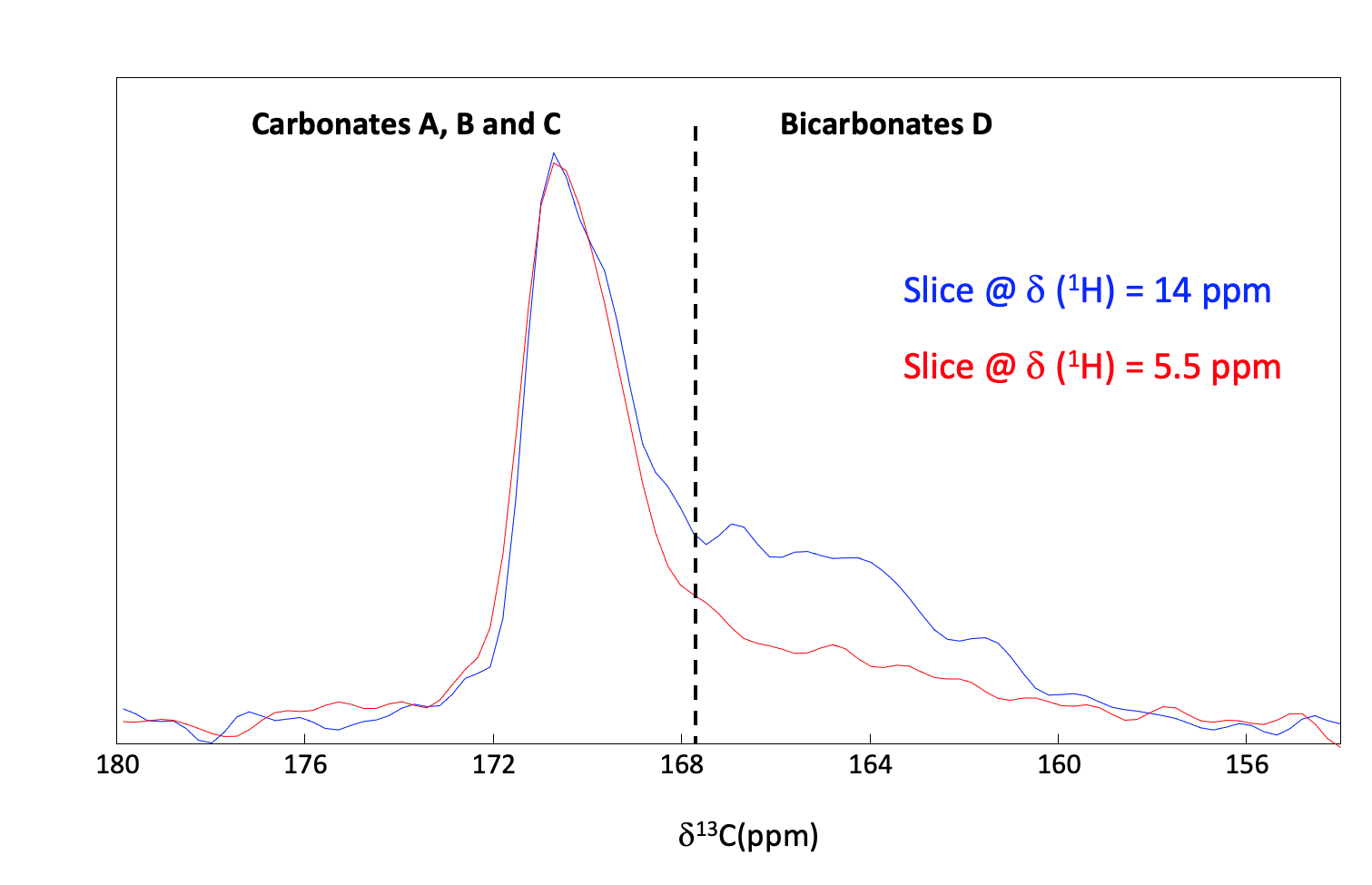


**Supplementary Figure 6.** ^13^C slices extracted from 2D ^1^H-^13^C HetCor spectrum of ^13^C-labelled *H. turberculata* larvae (72 hpf) displayed in **Supplementary Figure 5d**.

| **Resonance** | **A** | **B** | **C** | **D** |
| --- | --- | --- | --- | --- |
| **δ^13^C (ppm)** | 171.2 ± 0.1 | 170.5 ± 0.1 | 168.4 ± 0.1 | 164.0 ± 0.1 |
| ***Δ*_CSA_ (ppm)** | -57.5 ± 3 | -47 ± 3 | -53,3 ± 3 | +64 ± 3 |
| ***η*_CSA_** | 0.3 ± 0.1 | 0.4 ± 0.1 | 0.4 ± 0.1 | 0.8 ± 0.1 |
| **M_0_** | 3.7 ± 0.5 | 5.7 ± 0.5 | 2.1 ± 0.5 | 2.7 ± 0.5 |
| ***T*_CH_ (ms)** | 1.6 ± 0.1 | 0.8 ± 0.1 | 0.7 ± 0.1 | 0.35 ± 0.1 |
| ***T*_1_ρ (ms)** | - | 14.8 ± 1 | 5.5 ± 0.5 | 7.7 ± 0.5 |
| **Assignment** | CO_3_^2-^ | CO_3_^2-^ | CO_3_^2-^ | HCO_3_^-^ |

**Supplementary Table 3.** ^13^C NMR parameters obtained from **Supplementary Figure 5.** Spectrum seen in **Supplementary Figure 5b** is fitted using DMFit program and allows the extraction of the CSA parameters (Δ_CSA_ and η_CSA_) defined as follow:

Δ_CSA_ = δ_33_ - δ_iso_ and η_CSA_ = (δ_22_ - δ_11_) / (δ_33_ - δ_iso_)

with δ_iso_ = 1/3 (δ_11_+δ_22_+δ_33_) and the chemical shielding principal values δ_11_, δ_22_, δ_33_ ordered as follows: |δ_33_ - δ_iso_| ≥ |δ_11_ - δ_iso_| ≥ |δ_22_ - δ_iso_|.

Curves seen in **Supplementary Figure 5c** are fitted using classical I-S model through the following equation:

M(t_CP_) = M_0_ (1/(1 – T_CP_ / T_1_ρ(^1^H))) * (exp(-t_CP_/T_1_ρ(^1^H)) - exp(-t_CP_/T_CH_))

Where M_0_, t_CP_, T_CP_, T_1_ρ(^1^H) are the ^13^C CP intensity, the contact time, the CP dynamical parameter and the spin-lock relaxation time, respectively.


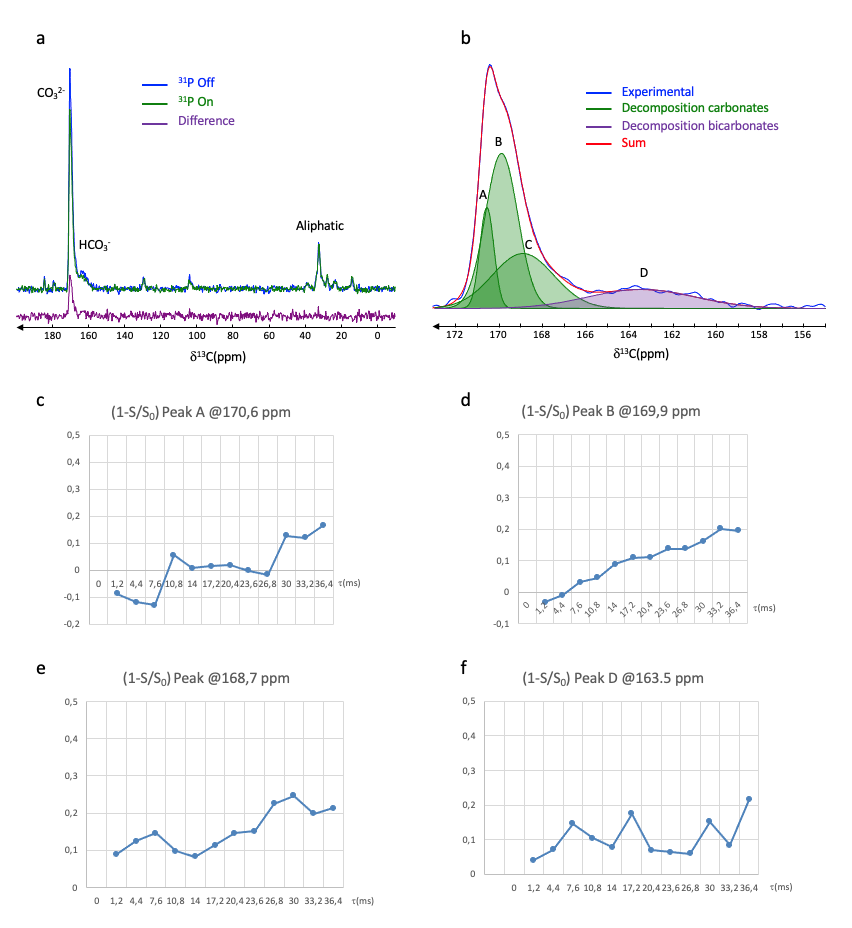


**Supplementary Figure 7.** (a) {^1^H}^13^C{^31^P} CP REDOR experiments for ^13^C-labelled *H. tuberculata* (72 hpf) recorded at 36.4 ms of dephasing time. Note the absence of dephasing for the aliphatic region. (b) Typical decomposition of the {^1^H}^13^C{^31^P} CP REDOR spectrum (^31^P off; τ = 36.4 ms). (c-f) REDOR curves for each ^13^C signals.


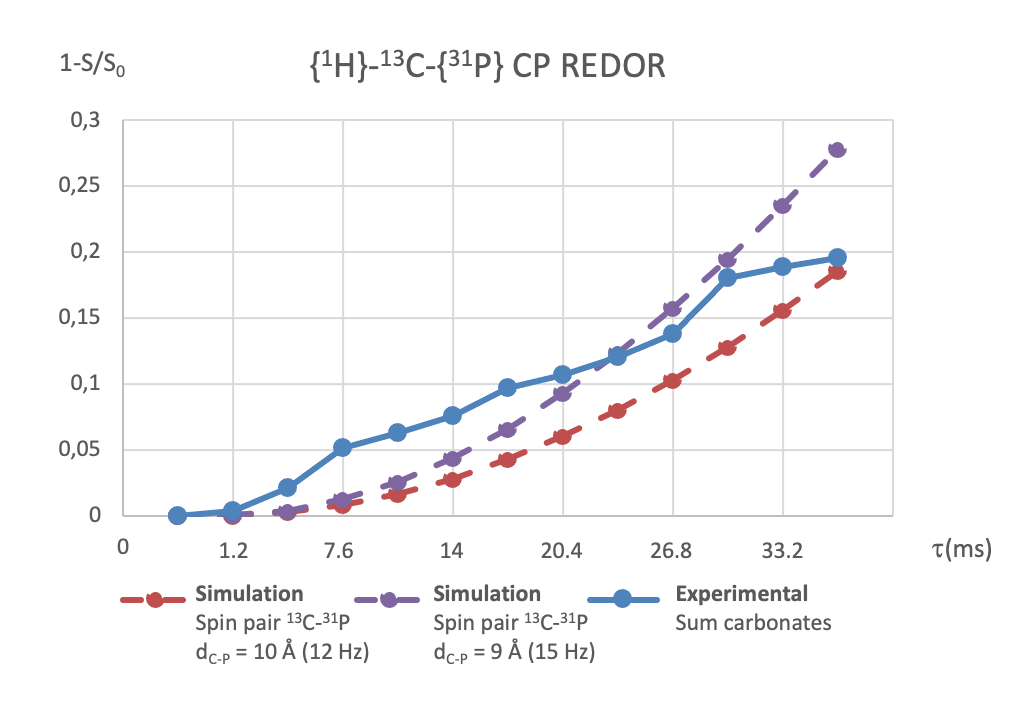


**Supplementary Figure 8.** {^1^H}^13^C{^31^P} CP REDOR experimental curve of the full carbonate resonance of ^13^C-labelled *H. tuberculata* (72 hpf) together with numerical simulation for one single ^13^C-^31^P spin pair at 10 Å (12 Hz) and 9 Å (15 Hz). If the end of the REDOR curve is somehow well described at long recoupling times but numerical simulation by a single ^13^C-^31^P spin pair fails to describe the rapid start.

**Supplementary Discussion 1.** The ^13^C CP MAS spectrum of ^13^C-labeled *H. tuberculata* 72 hpf larvae exhibits two distinct spectral regions (~171 and ~164 ppm, respectively) that can be properly fitted using four distinct resonances (named A, B, C and D; **Supplementary Figure 5a**). The carbonate/bicarbonate assignment is done through the analysis of slow spinning speed ^13^C CP MAS experiment, variable contact time ^13^C CP MAS experiment and 2D ^1^H-^13^C HetCor spectrum.

Carbonate and bicarbonate display close ^13^C NMR resonances and the assignment based on their ^13^C chemical shift can be ambiguous, even if bicarbonates usually exhibit lower δ^13^C than carbonates. However, it is now established that they can unambiguously distinguished based on their relative CSA parameters due to distinct symmetry.^1^ In particular, carbonates possess negative *Δ*_CSA_ whereas bicarbonates possess a positive one. The fitting of the ^13^C spinning side band manifold obtained from a slow spinning speed NMR experiment (**Supplementary Figure 5b**) allows the extraction of *Δ*_CSA_ and *η*_CSA_ parameters. As a result, resonances A, B and C possess a negative Δ_CSA_, varying from -45 to -57 ppm (exhibiting the most intense spinning side band on the left of the isotropic resonance), whereas resonance D exhibits a positive value, Δ_CSA_ ≈ +64 ppm (the most intense spinning side band on the right of the isotropic resonance) (**Supplementary Table 3**). Hence, resonances A, B, C are assigned to CO_3_^2-^ whereas resonance D is in the form of HCO_3_^-^.

Variable contact time ^13^C CP MAS experiments (**Supplementary Figure 5c**) is in agreement with this attribution. However, a clear distinction of carbonate species can be done based on their protonated environment as “slow” and “fast” CP regimes are observed: carbonate A exhibits a slow CP regime (*T*_CH_ = 1.6 ms) coherent with a depleted proton environment whereas carbonates B and C are characterized by a faster CP regime (*T*_CH_ = 0.8 and 0.7 ms, respectively) and a strong spin lock effect (*T*_1_ρ(^1^H) = 14.8 and 5.5 respectively). The proton environment around B and C carbonates is richer, probably with H_2_O and/or HCO_3_^-^ species closer than for carbonate A.

The 2D ^1^H-^13^C HetCor spectrum (**Supplementary Figure 5d**) confirms such findings as carbonate A correlates with H_2_O et HCO_3_^-^ whereas carbonates B and C correlates principally with HCO_3_^-^ (and with H_2_O to a lesser extend). As a consequence, carbonates B and C must be closer to HCO_3_^-^ than carbonate A.

Concerning bicarbonate D, the careful examination of their correlation is very instructive since a “banana” shape is observed characteristic of a strong disorder around these ions. A distribution of chemical shifts is observed both in the ^1^H (from 11 to 17 ppm) and the ^13^C dimension (from 160 to 168 ppm). According to previous numerical GIPAW calculations and comparison with experimental NMR data,^2^ for a given OH group, the strength of the H-bond increases with the δ(^1^H). As a result, bicarbonates of low ^1^H (~11 ppm) and ^13^C (160 ppm) chemical shifts, can be considered as rather “isolated” HCO_3_^-^, whereas bicarbonates of high ^1^H (~17 ppm) and ^13^C (168 ppm) chemical shifts, can be seen as “highly” H-bonded bicarbonates. In that latter case, the distance H•••CO_3_ increases with the H-bond strength, giving to bicarbonate a carbonate character in agreement with the increase of δ(^13^C) reaching the carbonate region (δ(^13^C) > 168 ppm).

This analysis is consistent with what has been observed for the study of disordered mineral environments in mature nacre from *H. tuberculata*.^3^ Similar ^13^C resonances had been detected and assigned similarly. The only difference concerns the larger line width for the larval shell. This seems to be consistent since one can imagine a more immature aragonite in the case of the larval shell rather than adult one.


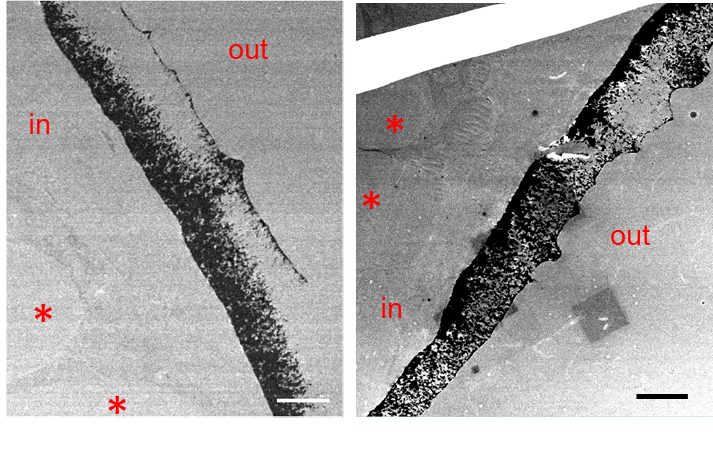


**Supplementary Figure 9.** STEM-HAADF images of two shell sections. The inner part of the shell is identified by the localization of the soft tissues (indicated by *). Scale bar = 2 µm.


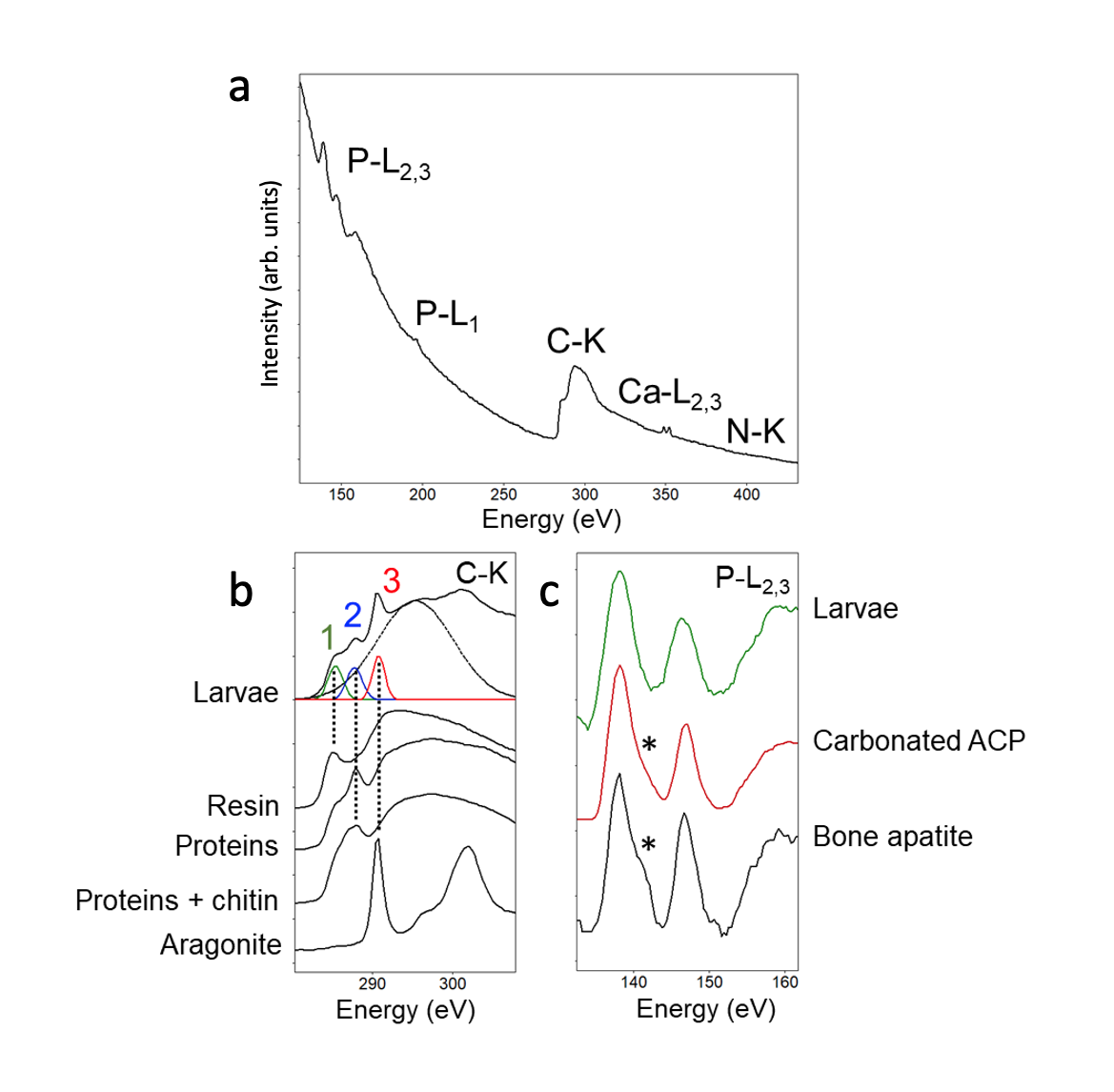


**Supplementary Figure 10.** (a) Typical EEL spectrum acquired on a shell section. The energy range corresponds to the phosphorus, calcium, carbon and nitrogen edges. (b) Carbon K-edge acquired on different samples. From top to bottom: a section of *H. tuberculata* larval shell (Larvae), embedding Resin, purified proteins extracted from larval shell (Proteins), a section of organic matrix from HT shell and aragonite. The spatial distributions of resin, organic species (proteins and chitin) and carbonate are obtained by fitting the peaks at 285 eV, 287 eV and 290 eV on C-K edge by three Gaussian functions (respectively green, blue and red curves labelled 1, 2 and 3). (c) Phosphorus L_23_-edge on larval shell compared to carbonated amorphous calcium phosphate (ACP) and bone apatite.

**Supplementary Discussion 2.** A typical EEL spectrum is presented in **Supplementary Figure 10a** for the energy range corresponding to the signal of phosphorus (L_1_ and L_23_-edges), carbon (K-edge), calcium (L_23_-edge) and nitrogen (K-edge). The energy scale of EEL spectra was calibrated using the peak maximum positions of the phosphorus and calcium L_23_ edges, respectively at 138 eV and 349 eV, according to XANES data from Cosmidis *et al*.^4^ Data were first processed to improve the signal-to-noise ratio by using principal component analysis (PCA) under Hyperspy, an open-source software suite (<http://hyperspy.org/>)^5^ as a filtering method. For each of the elemental edges (P, C, Ca and N), the background was removed by subtracting a power law model using Gatan’s Digital Micrograph software. Elemental maps were then built from the spectrum image acquired on a given area by integrating the intensity corresponding to each edge.

The carbon K- edge signal acquired at the different positions of the shell is composed of a series of peaks: a first narrow peak corresponding to 1s → π* electronic transitions is found at an energy between ~ 285eV and 290eV; a broader peak characteristic of 1s → σ* transitions is observed around 301 eV. The narrow peak position associated to the C 1s → π* transition varies with the chemical group determining the C bonding environment. Reference spectra were collected from different samples (**Supplementary Figure 10b**) to assign the fine features of the carbon edge to a given compound: in regions containing only embedding resin, the narrow peak is located at 285 eV (peak “1” in **Supplementary Figure 10b**); on purified proteins extracted from *H. tuberculata* shell and on a resin section of the organic matrix from *H. tuberculata* shell prepared as described in ref. 6, the maximum is centred around 287-288 eV (peak “2” in **Supplementary Figure 10b**). For aragonite, a peak assigned to the carbonate group is observed at 290 eV (peak “3” in **Supplementary Figure 10b**).^7^ A combination of these peaks was observed on *H. tuberculata* larva shell: the peaks “2” and “3” were respectively attributed to the presence of organic species and carbonate.

In order to map the different carbon bindings, a multi-Gaussian fitting was applied to the carbon peaks using the NLLS (non-linear least squares) method in the Gatan’s Digital Micrograph software. A typical example of multi-Gaussian fit is presented in **Supplementary Figure 10b**. For the spectrum images, the fit is repeated for each pixel across the scanned area, the Gaussian amplitude giving the relative abundance of each species.

Preparation of the reference samples was performed as follow: Chitin was nicely provided by France Chitine (Orange, France) and acidic soluble proteins were extracted from the shell through a classical procedure using 10% acetic acid for 4 days at room temperature.^6^


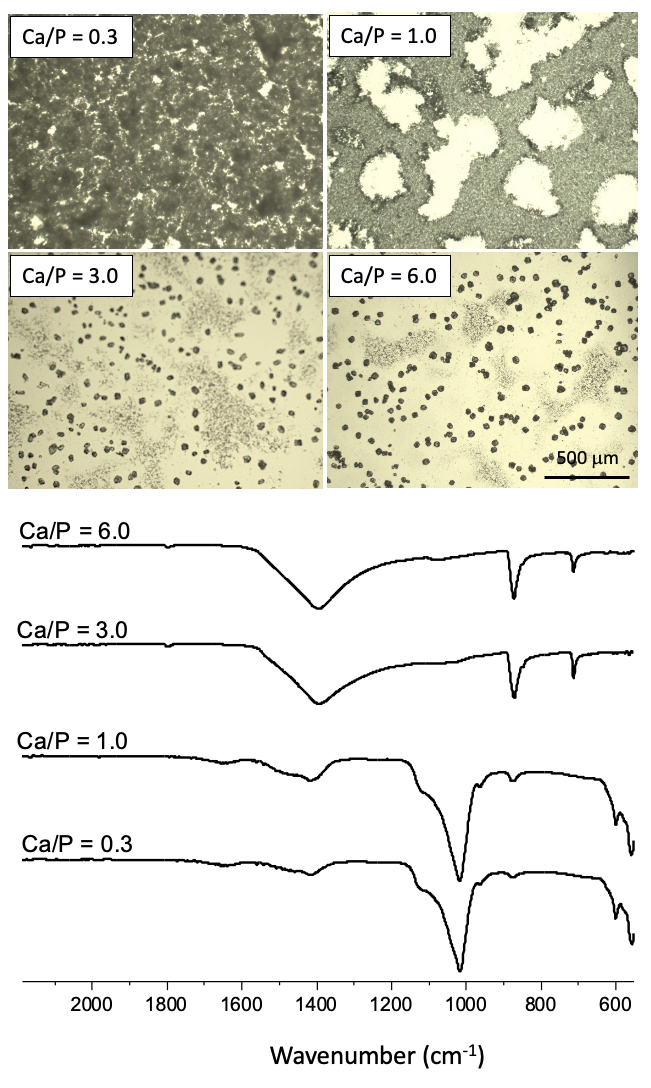


**Supplementary Figure 11.** Optical microscopy images and the corresponding FT-IR spectra showing the influence of the Ca/P ratio on the precipitation of the mineral phases after 3 days.

**References**

1. A. Di Bitetto, G. Kervern, E. André, P. Durand, C. Carteret, *J. Phys. Chem. C* **2017**, *121*, 6104.

2. C. Gervais, C. Coelho, T. Azaïs, J. Maquet, G. Laurent, F. Pourpoint, C. Bonhomme, P. Florian, B. Alonso, G. Guerrero, P. H. Mutin, F. Mauri, *J. Magn. Reson.* **2007**, *187*, 131.

3. W. Ajili, G. P. Laurent, N. Menguy, A. Gansmuller, S. Huchette, S. Auzoux- Bordenave, N. Nassif, T. Azais, *J. Phys. Chem. C* **2020**, *124*, 14118.

4. J. Cosmidis, K. Benzerara, N. Nassif, T. Tyliszczak, F. Bourdelle, *Acta Biomater.* **2015**, *12*, 260.

5. F. De La Peña, E. Prestat, V.T. Fauske, P. Burdet, T. Furnival, P. Jokubauskas, M. Nord, T. Ostasevicius, J. Lähnemann, K.E. MacArthur, D.N. Johnstone, M. Sarahan, J. Taillon, T. Aarholt, Pquinn-Dls, V. Migunov, A. Eljarrat, J. Caron, S. Mazzucco, B. Martineau, Suhas Somnath, T. Poon, T. Slater, C. Francis, Actions- User, M. Walls, N. Cautaerts, N. Tappy, F. Winkler, G. Donval, *Hyperspy/Hyperspy: Release v1.6.2*, Zenodo**2021**.

6. N. Gehrke, N. Nassif, N. Pinna, M. Antonietti, H. S. Gupta, H. Cölfen, *Chem. Mater.* **2005**, *17*, 6514.

7. J. A. Brandes, S. Wirick, C. Jacobsen, *J. Synchrotron Radiat.* **2010**, *17*, 676.
